# Neural and Molecular Mechanisms Involved in Controlling the Quality of Feeding Behavior: Diet Selection and Feeding Patterns

**DOI:** 10.3390/nu9101151

**Published:** 2017-10-20

**Authors:** Tsutomu Sasaki

**Affiliations:** Laboratory for Metabolic Signaling, Institute for Molecular and Cellular Regulation, Gunma University, 3-39-15 Showa-machi, Maebashi, Gunma 371-8512, Japan; tsutomus@gunma-u.ac.jp; Tel.: +81-27-220-8846

**Keywords:** food preference, diet choice, macronutrient selection, feeding rhythm

## Abstract

We are what we eat. There are three aspects of feeding: what, when, and how much. These aspects represent the quantity (how much) and quality (what and when) of feeding. The quantitative aspect of feeding has been studied extensively, because weight is primarily determined by the balance between caloric intake and expenditure. In contrast, less is known about the mechanisms that regulate the qualitative aspects of feeding, although they also significantly impact the control of weight and health. However, two aspects of feeding quality relevant to weight loss and weight regain are discussed in this review: macronutrient-based diet selection (what) and feeding pattern (when). This review covers the importance of these two factors in controlling weight and health, and the central mechanisms that regulate them. The relatively limited and fragmented knowledge on these topics indicates that we lack an integrated understanding of the qualitative aspects of feeding behavior. To promote better understanding of weight control, research efforts must focus more on the mechanisms that control the quality and quantity of feeding behavior. This understanding will contribute to improving dietary interventions for achieving weight control and for preventing weight regain following weight loss.

## 1. Introduction

The numbers of overweight and obese individuals have been increasing worldwide [1]. In 2015, diabetes and obesity were ranked the third and fourth leading health risk factors, respectively, in the world [2]. Both type 2 diabetes and obesity have significant negative impacts on the human lifespan [3]. Diabetes and obesity are treated through lifestyle interventions (dieting and exercise) and medical interventions (pharmacotherapy and surgery) [4]. Although lifestyle interventions cost less than medical interventions, low patient compliance hinders efficacy. To reduce the cost of health care, it is necessary to improve the efficacy of lifestyle interventions. To that end, it is useful to gain a better understanding of the mechanisms that regulate feeding behavior.

Doctors have been instructing patients in healthful eating since the medieval era, by emphasizing “what, when, and how much” people should eat. The content, the timing, and the quantity of meals are important factors to take into consideration. These factors are important determinants of obesity, though the ideal macronutrient composition for maintaining a healthy weight remains unclear. Feeding behavior modifications, such as altering meal timing and macronutrient composition, have been considered as novel approaches for achieving weight loss and preventing weight regain [5]. Following a healthy dietary pattern has been associated with less weight regain [6]. Moreover, eating patterns and food choices were important determinants of weight loss after Roux-en-Y gastric bypass surgery [7]. Conversely, weight cycling was associated with an elevated fat preference in obese women and female rats [8,9]. Therefore, for successful weight loss and weight maintenance, what and when we eat, in addition to how much we eat, needs to be taken into account.

The neural and molecular bases of individual differences in the selection, ingestion pattern, and proportioning of specific macronutrients are not fully understood. Current research on the central regulation of body weight is mainly conducted from the perspective of energy balance (energy intake vs. expenditure). This approach has unveiled many molecular mechanisms and the neural circuitry that contributes to weight control [10]. However, this research approach considers caloric intake (quantity), the default parameter to be measured. Thus, attention has been diverted from deciphering the neural and molecular mechanisms involved in controlling the qualitative aspects of feeding, namely, macronutrient selection and feeding patterns. Consequently, the mechanisms that regulate macronutrient selection and feeding patterns remain poorly understood.

The purpose of this review was to identify the knowledge gaps currently in the literature that hinder a full understanding of the central mechanisms that regulate macronutrient selection and feeding patterns. To that end, I summarized the current knowledge of these processes involved in controlling weight and health. I included evidence obtained from both human and animal models. I focused on summarizing current knowledge about the central regulation of feeding behavior, which is mostly based on animal studies. This review is expected to promote research on these knowledge gaps, which can lead to determining the precise mechanisms that regulate these processes.

## 2. Basic Concepts in the Regulation of Feeding Behavior

**Figure 1:**
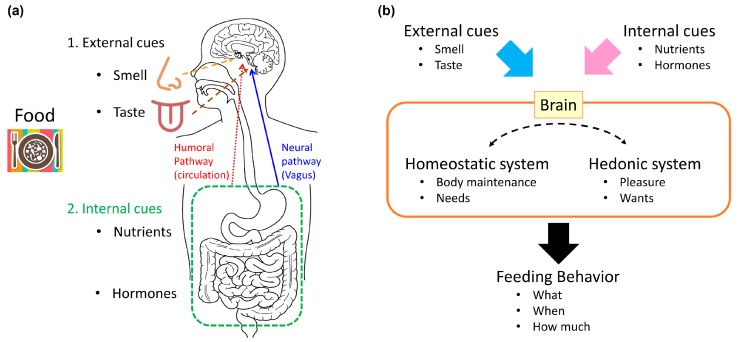
Basic concepts in feeding regulation: (**a**) The types of informational cues conveyed to the central nervous system; (**b**) Two systems in the brain integrate information to regulate feeding behavior.

Feeding behavior is a complex process. It incorporates homeostatic need, hedonic pleasure, and higher cognitive processes, such as contextual and learned information. Animals eat, at least to satisfy their homeostatic need for calories, and they select food to address their needs for nutrients that cannot be biosynthesized. In the central nervous system (CNS), homeostatic and hedonic systems process information transmitted from the periphery, which reflects peripheral needs and the nature of food (either food already ingested or food to be ingested), and they use this information to control feeding behavior [11]. Animals determine their feeding behaviors, based on the integration of external cues (such as smell and taste), internal cues, that reflect internal metabolic status and homeostatic needs (such as nutrients and hormones), motivation, and experience [10,12] (Figure 1).

Before discussing the mechanisms that regulate macronutrient selection and feeding patterns, the basic concepts of feeding behavior regulation are summarized below to provide a general understanding of feeding behavior research. For more details on appetite control circuits, please refer to an excellent review, recently published by [13].

### 2.1. Information Conveyed to the Central Nervous System

Before food is ingested, both smell and taste cues are sent to the CNS, and these influence feeding behavior. Once food enters the gastrointestinal tract, the post-ingestive effects of food are mediated by nutrient and humoral factors that respond to the digested nutrients. Post-ingestive nutrient information is conveyed from the gut to the brain, via two pathways: the neural pathway and the humoral pathway [10].

The neural pathway is mediated by the vagal afferents that innervate the gastrointestinal tract and hepatic portal vein [13,14]. The vagal afferents are stimulated by nutrients, hormones, and the food-induced mechanical stretch of the intestines; then, vagal neural transmission activates the nucleus of the solitary tract (NTS) in the brainstem. NTS activation is important for satiation. Information conveyed to the NTS is relayed to other CNS nuclei, which are important for feeding regulation, such as the parabrachial nucleus (PBN), the paraventricular nucleus (PVN), and the arcuate nucleus (ARC) of the hypothalamus.

The humoral pathway is mediated by nutrients and hormones [1]. The basic components of macronutrients, such as glucose, amino acids, and fatty acids, can serve directly as nutrient signals to the brain. Nutrients also affect the release of several hormones that are important for feeding regulation, such as leptin, insulin, and ghrelin. Leptin is an adipokine, crucial for regulating feeding behavior and body weight [15]. The long isoform of the leptin receptor is expressed in several brain nuclei, including the ARC, the PVN, the dorsomedial nucleus of the hypothalamus (DMH), the lateral hypothalamic area (LH), and ventromedial nucleus of the hypothalamus (VMH) [16]. In rodents, a homozygous loss-of-function mutation in the leptin receptor causes hyperphagia, obesity, and diabetes [17,18]. Insulin is a hormone secreted from pancreatic beta cells, which acts to lower blood glucose levels. Insulin acts on peripheral tissues, such as the liver, skeletal muscle, and adipose tissue, to cause anabolic effects. In addition, the insulin receptor is expressed widely in the CNS [19], and central insulin receptors mediate the catabolic effects of insulin (which suppresses feeding) [20]. Both leptin and insulin regulate pro-opiomelanocortin (POMC) and agouti-related peptide (AgRP) neurons, located in the ARC, at multiple levels; in these neurons, leptin and insulin regulate transcription, peptide processing, and synaptic transmission. These hormones serve as satiety signals to regulate feeding behavior [1]. Ghrelin is a peptide hormone, secreted from the stomach; it functions as a hunger signal [21]. Circulating ghrelin levels are highest before a meal, and they fall with feeding [22]. Ghrelin stimulates the vagal afferent nerves, to send information to the hypothalamus through the brainstem [23,24,25]. Ghrelin was also proposed to stimulate AgRP neurons directly in the ARC to promote feeding [26,27,28].

### 2.2. Homeostatic System

The center for the homeostatic control of appetite is located in the hypothalamus, which integrates energy information conveyed from the periphery [1,13] and neural information transmitted from the NTS. The humoral information related to feeding is directly sensed by the primary feeding center, the ARC. The ARC is located close to the median eminence, which permits entry of circulating humoral factors into the brain [29].

There are two major neuronal subtypes in the ARC: anorexigenic POMC neurons and orexigenic AgRP neurons [30]. Both these neurons receive nutritional information from the periphery—satiety signals stimulate POMC neurons and inhibit AgRP neurons. Conversely, hunger signals stimulate AgRP neurons [31], and AgRP neurons transmit inhibitory gamma-aminobutyric acid (GABA)-ergic signals to POMC neurons. POMC neurons and AgRP neurons generally project to the same targets in secondary centers for satiety (PVN, VMH, and PBN) and for hunger (LH) [30]. Target neurons express melanocortin type 4 receptors (MC4Rs), which are influenced by the POMC product, alpha-melanocyte-stimulating hormone (α-MSH), and by AgRP (an inverse agonist). The MC4R is a Gs-type G protein-coupled receptor (GPCR); thus, MC4R activation raises intracellular cyclic AMP (cAMP) levels. The balance between the activities of anorexigenic POMC neurons and orexigenic AgRP neurons dictates the activity of the secondary CNS satiety center. POMC, AgRP, and MC4R comprise the central melanocortin system, and mutations in this system are most frequent among patients with the monogenic form of human obesity [32].

Orexigenic AgRP neurons also produce neuropeptide Y (NPY), an orexigenic neurotransmitter. Two forms of NPY receptors (Y1R and Y5R) are expressed in target NPY/AgRP/GABA neurons in the secondary satiety center [33]. Activation of Y1R or Y5R, both Gi-type GPCRs, reduces the intracellular cAMP levels. Therefore, both AgRP and NPY induce consistent changes in intracellular secondary messengers that inhibit target neuron activity.

In general, orexigenic neurons are stimulated by hunger signals, and anorexigenic neurons are stimulated by satiety signals.

### 2.3. Hedonic System

The hedonic regulation of appetite comprises several components, including appetitive desire (motivation, incentive salience, “wanting”) and consummatory enjoyment (pleasure, reward value, “liking”), and learning [34,35]. Some of these concepts can be attributed to particular biological systems. For example, motivation arises from the dopamine system, pleasure arises from the opioid system and learning arises from the hippocampus (Table 1). Although these assignments are somewhat oversimplified, they are instructive for grasping the concepts of feeding control.

#### 2.3.1. Dopamine System

The hedonic drive to eat is regulated by the activity of the mesolimbic dopamine system [10]. Dopamine neurons, located in the ventral tegmental area (VTA), project to the nucleus accumbens (NAc). Dopamine released in the NAc plays an important role in motivated behavior [36]. The mesolimbic dopamine system also promotes learning associations between natural reward and the environment [37,38]. Consumption of palatable food increases the firing of VTA dopamine neurons, which leads to increased dopamine release in the NAc [39]. Thus, behavior involved in approaching food is primed by rapidly increasing the excitatory input to the VTA dopamine neurons [40]. The ingestion of palatable food also causes dopamine release in the striatum and activates reward circuitry in humans, in proportion to the rating of meal pleasantness [41,42].

Dopamine signaling is modulated by brain areas involved in food selection and decision-making, such as the medial prefrontal cortex and the orbitofrontal cortex. These areas process information about our external environment, assign reward values to food cues, and inform us on the availability and attractiveness of food.

The dopamine system is also regulated by internal homeostatic signals that are conveyed by hormones and nutrients. Humoral factors can directly influence neurons in the dopamine system, or indirectly influence them, by signaling through hypothalamic or brainstem neurons [43]. Neurons in the VTA and/or NAc, express receptors for leptin [44,45], insulin [44], glucagon-like peptide-1 (GLP-1) [46], ghrelin [47,48], and orexin [49]. Hormones that convey satiety signals, such as leptin [50], insulin [51,52], and GLP-1 [46], inhibit the activity of dopamine neurons and/or reduce dopamine release in the NAc. In contrast, hormones that convey hunger signals, such as ghrelin [47,53], orexin [54,55], and NPY [56], have the opposite effects on the dopamine system. A human magnetic resonance imaging study reported that fasting was associated with sensitization of the striatal reward system to the anticipation of food reward, irrespective of reward magnitude, and ghrelin signaling increased neural reactivity during the expectation of food-related reward [57].

#### 2.3.2. Opioid System

Endogenous opioids also modulate some aspects of food reward. Endogenous opioid peptides include endorphins, enkephalins, dynorphins, and nociceptins, which act through four opioid receptors, known as mu (MOR), delta (DOR) and kappa (KOR) opioid receptors, and nociception receptors [58,59]. Beta-endorphin and enkephalins are particularly important for food-related reward, but these two ligands have markedly different distributions and expression levels within the nervous system [60]. Enkephalins are distributed widely throughout the brain, including the NAc, the ventral pallidum (VP), the amygdala, and the PBN. In contrast, the central source of β-endorphin is mostly POMC neurons in the ARC, and these neurons project to very limited sites, including the PVN and NAc [61,62]. Enkephalins act through MORs and DORs, but β-endorphin acts only through MORs. Dynorphins act through KORs, and nociceptin acts through the nociception receptor.

Systemic opioid antagonist administration prevents the formation and expression of taste preference [63,64,65,66]. Opioid receptor blockade reduces the perceived pleasantness of palatable foods, without significantly altering feelings of hunger, basic taste perception, or taste intensity [67,68,69]. Thus, MOR stimulation in the NAc induces a strong hyperphagic drive for palatable foods [70,71,72,73], but infusion of MOR antagonists into the NAc decreases palatable food intake [74].

Although opioids modulate the palatability of food [75], enkephalins can impact the motivational properties of food [76,77]. One study compared feeding behaviors between mice with a genetic knockout of the proenkephalin gene and mice with a β-endorphin deficiency. They revealed that endogenous enkephalins primarily set a background motivational tone for feeding behavior, but β-endorphin signaling was specifically involved in feeding driven by palatability, as opposed to incentive-driven motivation [78]. MOR hotspots in the NAc and the VP contribute to both the hedonic impact of food reward (“liking”) and the incentive motivation (“wanting”) for seeking food and other rewards [79]. Therefore, the opioid system influences feeding behavior by affecting both the hedonic impact and the motivational aspects of food.

### 2.4. Concluding Remarks on the Regulation of Feeding Behavior

The neural and molecular mechanisms that control feeding behavior integrate sensory cues (smell, taste, interoception, etc.) and humoral cues (nutrients, metabolites, and hormones) through homeostatic and hedonic mechanisms. However, how these systems process information gathered from the various modalities and the extent of the neural circuits that constitute each system are not fully understood. Furthermore, although it is evident that the two systems interact reciprocally, the extent of reciprocal interactions and the exact mechanisms involved in these interactions remain elusive.

In this section, I did not cover the importance of the visual cues, partly because rodent models are not optimal for studying this feature of feeding behavior. Visual information, such as what the food looks like and the feeding environment (where you eat), can evoke past memories of feeding and influence feeding behavior. Further study is needed to investigate how visual information fits into the multimodal regulation of feeding behavior.

## 3. Macronutrient-Based Diet Selection (What We Eat)

Food preferences are prominent determinants of food intake in humans. Food preference was positively correlated with reported food intakes [80,81], and it is the first factor to influence the choice of food in developed countries [82]. Food preferences are influenced by personal experiences (e.g., food exposure during childhood) [83], economics [84], and genetic factors, particularly related to taste perception [85,86]. One study that analyzed a UK twin cohort reported a strong genetic influence on preferences for meat and distinctive-tasting foods [87]. Therefore, diet selection is influenced by both innate genetic mechanisms, which are conserved among humans (and possibly across multiple species), and experience-based acquired mechanisms, which are different among individuals. This review focuses on the conserved mechanisms, i.e., macronutrient selection effects on health, and macronutrient selection regulation at the molecular and neurocircuitry levels.

### 3.1. Importance of Macronutrient Selection for Controlling Weight and Health

“Eating healthy” often means ingesting well-balanced macronutrients (and also micronutrients), in addition to eating proper amounts. Macronutrient composition is known to modify endocrine signals [88,89,90] and modulate brain neurotransmitter dynamics [91,92,93,94]. In addition, meal sequences have a significant impact on post-prandial glycemic control through endocrine signaling. For example, eating meat or fish (protein) before eating rice (carbohydrate) enhances GLP-1 secretion, delays gastric emptying, and ameliorates post-prandial glucose excursions in healthy humans and individuals with type-2 diabetes [95].

Excessive intake of a fat-rich or sugar-rich diet is detrimental to health. However, there have been long-standing debates on the health benefits of fat restriction versus carbohydrate restriction [96,97,98]. Fat restriction was reported to reduce adiposity over the long term, but not body weight, compared to carbohydrate restriction [96]. Meta-analyses of controlled isocaloric feeding studies also indicated that, to increase energy expenditure and reduce adiposity, fat restriction is favored over carbohydrate restriction [97]. Mixed results have been reported on the effects of carbohydrate restriction, presumably because the type of fat consumed to sustain caloric intake affects health. When more unsaturated fatty acids were used to replace carbohydrates, carbohydrate restriction achieved better glycemic control and lipid profiles compared to isocaloric fat restriction, but both diets achieved the same degree of weight loss [98]. Systemic meta-analyses of randomized control trials also showed that replacing carbohydrates with unsaturated fatty acids improves glycemic control [99]. A recent prospective cohort study on dietary intake indicated that high carbohydrate intake is associated with a higher risk of total mortality; indeed, isocaloric replacement of carbohydrate with poly-unsaturated fatty acids was associated with an 11% lower risk of mortality [100]. Based on that evidence, the most recent dietary guidelines no longer restrict the percentage of calories to be derived from fat in the diet (2015–2020 Dietary Guidelines for Americans) [101].

### 3.2. Mechanisms That Regulate Macronutrient Preference

Among the macronutrients, both fat and carbohydrates (particularly simple sugars) are deemed highly rewarding. However, macronutrient selection behavior cannot be fully understood by studying only hedonic feeding, because sometimes a person must select between a diet rich in fat and a diet rich in simple sugars. This quandary leads to the questions: how do we choose what we eat and what is known about the mechanisms that regulate macronutrient-based diet selection?

#### 3.2.1. Humoral Factors that Influence Macronutrient Preference

Fibroblast growth factor 21 (FGF21) is the only known hormone to date that regulates macronutrient-specific preference. FGF21 regulates carbohydrate preference [102,103]. FGF21 is secreted in response to various metabolic stresses [104]. Although FGF21 is secreted from various tissues, secretion from the liver determines the circulating FGF21 level. Ingesting simple sugars activates the hepatic carbohydrate-responsive element-binding protein (ChREBP), which increases production of FGF21 from the liver [102]. Circulating FGF21 peaked 2 h after an oral sucrose challenge in humans [105], and a 3-day carbohydrate-rich feeding protocol caused an 8-fold increase in plasma FGF21 levels, compared to a eucaloric control diet, in healthy men [106]. Circulating FGF21 activated hypothalamic PVN neurons and decreased sweet preference [102,103]. Therefore, FGF21 serves as a negative feedback signal to the CNS and regulates carbohydrate preference.

Ghrelin is another peptide hormone that may modulate feeding behavior in a macronutrient-specific manner. Ghrelin is secreted from the stomach, and it promotes food intake [21]. Central administration of ghrelin by intracerebroventricular (icv) injection was reported to preferentially enhance fat intake over carbohydrate intake under a paradigm of two food choices, in both high carbohydrate-preferring rats and high fat-preferring rats [107]. However, in another study that used a three food choice paradigm (chow, sucrose, and lard), icv-injected ghrelin increased both chow and lard intakes; moreover, an injection of ghrelin into the VTA increased chow intake without affecting sucrose or lard intake [108]. Although ghrelin has been shown to target reward areas and to increase motivated behaviors for fat [109] and sucrose [53,110], its effect on macronutrient preference may be context-dependent. Therefore, the role of ghrelin in regulating macronutrient preference is controversial.

#### 3.2.2. Homeostatic Feeding Mechanisms that Influence Macronutrient Preference

The central melanocortin system is the most important system for the homeostatic control of energy balance, and it regulates the quantity of feeding. However, genetic studies have pointed out that it is also important for controlling macronutrient preferences (Table 2). Individuals with null mutations of *MC4R* showed a high fat preference, high intake of a high-fat diet (HFD), a reduced carbohydrate preference, and reduced intake of a high-sucrose diet [111]. These individuals found the high-sucrose diet less palatable than the HFD, but they rated the palatability of a HFD similar to ratings from a control group [111]. *Pomc*-null mice, which lack the agonist for MC4R, showed an elevated fat preference [112]. Yellow agouti (*Ay/a*) mice, which overexpress the inverse agonist for MC4R, also showed an elevated fat preference, and in addition, a reduced carbohydrate preference [113]. Therefore, loss-of-function mutations in the central melanocortin system consistently increased fat preference and reduced carbohydrate preference, in rodents and humans.

Several other neuropeptides have been reported to regulate preference in a macronutrient-specific manner. Fat preference was increased by galanin and reduced by neuromedin U (NMU); sucrose preference was increased by neuropeptide Y (NPY) and reduced by oxytocin (Oxt). However, conflicting results were reported from studies on the roles of melanocyte concentrating hormone (MCH) and corticotropin releasing hormone (CRH) on macronutrient preference.

Galanin injections into the PVN of rats caused a preferential increase in the consumption of a fat diet, when single-macronutrient diets (protein, carbohydrate, or fat) were presented [114]. In a macronutrient choice scenario, *galanin* knockout mice consumed significantly less fat [115], and conversely, transgenic mice that overexpressed galanin consumed significantly more of a fat-rich diet, without any change in sucrose preference [116]. In the hypothalamus, galanin stimulates release of the opioid, enkephalin, throughout the brain, which promotes fat preference [117]. Ingestion of a HFD caused an increase in the expression, production, and release of galanin in the anterior PVN of the hypothalamus [118]. Conversely, high-carbohydrate meal ingestion caused a reduction in galanin expression in the PVN [119]. Those findings indicated that fat ingestion promoted galanin release and fat preference through a positive feedback mechanism.

A few reports suggested that NMU decreased fat preference. Peripheral administration of NMU in rats reduced operant responding for high-fat food [120]. Conversely, a genetic knock-down of the high-affinity receptor for NMU (NMU receptor 2) in the rat PVN increased preference for high-fat foods without affecting standard chow intake [121].

NPY was shown to be orexigenic, because it promoted caloric intake. In addition, NPY promoted carbohydrate preference. Ingestion of carbohydrate and sucrose were preferentially increased with NPY administration, either with injections into the PVN [122] or with icv injections [123]. Variants in NPY receptors 1 and 5 were associated with a lower carbohydrate intake, characterized by a lower consumption of mono- and disaccharides [124]. High-carbohydrate meal consumption increased NPY expression and NPY immunoreactivity in the ARC and PVN of the hypothalamus, but not in other hypothalamic areas [119]. However, when rats were allowed free choice among foods composed of saturated fat, 30% sucrose solution, and standard chow, bilateral administration of NPY in the NAc increased fat intake, but not sugar or chow intake. This response was mediated by Y1R [125]. Therefore, although NPY predominantly promoted carbohydrate intake by acting on the PVN, it also promoted preferential intake of fat in the NAc.

Oxt is a well-known pituitary peptide hormone produced in the PVN and supraoptic nucleus of the hypothalamus. Oxt is secreted into the hypophyseal portal vein from the posterior pituitary (neurohypophysis), and it exerts systemic effects [126]. Oxt is also secreted within the CNS, where it functions as a neurotransmitter and affects social memory [126]. Moreover, Oxt suppresses sucrose and carbohydrate intake and preference [127]. Oxt knockout mice showed an enhanced sucrose preference, but no change in fat preference [128,129,130,131]. The Oxt receptor inhibitor, L-368,899, stimulated carbohydrate intake [132]. Fluctuations in sugar intake over the menstrual cycle in humans inversely correlated with blood Oxt levels; sugar intake was low during ovulation, when Oxt was high, and high during the luteal phase, when Oxt was low [133,134]. The Oxt receptor, a Gq-type GPCR, is widely distributed in the CNS; it is detected in the hypothalamus, VTA, NAc, amygdala, and spinal cord [135]. Thus, Oxt could regulate food preference at several locations in the brain. Intranasal Oxt administration suppressed hypothalamic activation in response to visual food cues [136]; Oxt action in the VTA affected sucrose intake [137]; and Oxt infusion into the NAc reduced palatable sucrose consumption [138]. Peripheral Oxt may modulate sweet taste sensitivity, because the Oxt receptor is also expressed in tastebud cells [139]. In addition, an intraperitoneal injection of Oxt, which elevated plasma Oxt levels, was sufficient to reduce sweet taste sensitivity in mice [140]. Sucrose or carbohydrate intake also stimulated Oxt neuronal activity—sucrose-fed mice exhibited two-fold higher c-Fos (+) activity in PVN Oxt neurons than intralipid-fed mice [131]. Moreover, Oxt expression increased after the ingestion of sucrose and cornstarch, but not saccharin [131,132]. Therefore, Oxt regulates sucrose and carbohydrate intake through a negative feedback system.

MCH was proposed to mediate the post-ingestive reward effect of sugars, because MCH-treated rats showed significant increases in the ingestion of sucrose and glucose solutions, but not saccharin [141]. Hypothalamic MCH neuronal activity was proposed to transmit the nutrient value of sugar. Optogenetic activation of MCH neurons was sufficient to invert the normal preference of sucrose over sucralose (an artificial sweetener lacking the post-ingestive effect of sugar) by increasing striatal dopamine levels. Ablation of MCH neurons reduced striatal dopamine release upon sucrose ingestion and abolished the preference for sucrose over sucralose [142]. However, mice with a genetic knockout of MCH receptor 1, the only MCH receptor in rodents, showed no impairment in glucose-conditioned flavor preferences [143]. Thus, the role of MCH in regulating carbohydrate preference remains controversial.

It is not clear what roles CRH and the hypothalamic-pituitary-adrenal (HPA) axis play in macronutrient preference regulation, because studies have reported inconsistent results. Although these pathways may modulate food preference, the effect may depend on the degree of activation. For instance, an icv injection of CRH (0.5 μg) significantly increased the consumption of a saccharin solution in rats, but a ten-fold larger dose abolished saccharin intake [144]. Administration of exogenous glucocorticoids had differential effects on food preferences. One group reported that an adrenalectomy (ADX) in rats significantly attenuated the ingestion of all three macronutrients over 24 h, but during the early dark cycle, when circulating corticosterone (CORT) levels normally peaked, ADX had the strongest suppressive impact on carbohydrate ingestion [145]. They further showed that a CORT injection in ADX–treated rats preferentially stimulated carbohydrate ingestion [146]. Another group reported that an ADX in rats did not affect carbohydrate or fat intake, but when rats self-administered CORT in a drinking solution (40 μg/mL), fat intake increased [147]. These data suggested that a large dose of CORT promoted a fat preference and a relatively lower dose of CORT promoted carbohydrate consumption. Chronic mild stress, which increased the activity of the HPA axis, was reported to increase fat preference in mice [148] and decrease sucrose preference in rats [149]. Injections (icv) of CRH (0.05 and 0.5 μg) were reported to attenuate the consumption of a novel food choice, without affecting the intake of familiar foods [150]. Thus, the roles of CRH and the HPA axis in regulating food preferences may depend on the degree of activation, and not on a specific macronutrient. Controlling the stress level may be required in experiments that specifically address macronutrient-specific effects of CRH on food preference.

The relationships between neuropeptides and macronutrients are summarized in Table 3.

#### 3.2.3. The Hedonic System and Macronutrient Preference

Nutrient sensing by the hedonic system is important for regulating food preference. Nutrients, particularly glucose-containing sugars and fat, have potent reward values, and they promote dopamine release in the NAc [43,151]. Both the orosensory sweet taste stimulus and the post-ingestive effect of sugar stimulate the NAc. Sweet taste information sensed in the taste papillae is conveyed to the NTS, then to the PBN, the insular cortex, and the NAc [152,153]. The importance of the post-ingestive effect of glucose on dopamine release in the NAc was determined with the direct administration of glucose into the stomach [154]. A sweet taste preference or flavor-nutrient conditioning can be induced, even in the absence of sweet taste perception, when the post-ingestive sensing mechanisms were allowed sufficient time to take effect [155]. Glucose sensing in the hepatic portal vein triggered dopamine release in the NAc [156,157], thus vagal afferents transmitted the post-ingestive effect of glucose to the dopamine system. Multiple hypothalamic circuits also sense glucose [158], and glucose can also regulate the dopamine system through hypothalamic MCH neurons in the LH [142]. Therefore, peripheral and central glucose monitoring systems influence the dopamine system and modulate food preference. Fat sensing also influences the dopamine system. Delivery of small amounts of triglycerides to the brain through the carotid artery abolished the preference for palatable food and reduced the motivation to engage in food-seeking behavior. In contrast, targeted disruption of the triglyceride-hydrolyzing enzyme, lipoprotein lipase, specifically in the NAc, increased the palatable food preference and food-seeking behavior [159].

The role of the dopamine system in reward is evident, but its role in the macronutrient-specific regulation of food preference is not clear. Different groups have reported mixed results. Systemic antagonists of dopamine D1 and D2 receptors blocked the acquisition and expression of sugar-conditioned flavor preference, but not fat-conditioned flavor preference [160]. That finding indicated that the dopamine system was not the sole regulator of fat preference. However, in obese rats, gastric bypass surgery activated the gut peroxisome proliferator activated receptor alpha (PPARα)–striatal D1 receptor pathway, which reduced fat intake and preference [161]. In *Drosphila*, protein hunger was encoded by the branch-specific plasticity of a bifunctional dopamine circuit [162]. Collectively, these findings suggested that the dopamine system may contribute to the reward effects of nutrients in each context, but it may not encode the preference toward a particular macronutrient.

The MOR was implicated in promoting the intake of a HFD. Genome-wide association studies (GWAS) identified a locus of fat intake; the gene that encodes the MOR (*OPRM1*) was associated with fat intake in an independent sample of 490 young adults [163]. In rats, an intraperitoneal injection of morphine (a MOR agonist) was given under a self-selection feeding paradigm, with carbohydrate, protein, and fat simultaneously available. That study showed that MOR was associated with an increase in total caloric intake and increased preferences for fat and protein [164]. When rats were given a choice between fat and carbohydrate diets, MOR stimulation within the NAc preferentially stimulated fat ingestion without any effect on carbohydrate ingestion [165]. MOR stimulation in the NAc activated various hypothalamic areas, the VTA, the substantia nigra, and the NTS [70]. In mice, administration of non-selective and mu-selective opioid receptor antagonists inhibited the preference for fat over sucrose [166]. Subcutaneous administration of the non-selective opioid receptor antagonist, naltrexone, reduced the acquisition of fat reinforcement, but not the expression of learned reinforcement [167]. In rats, a roux-en-Y gastric bypass markedly suppressed fat intake and preference, and downregulated MOR protein levels in the striatal and prefrontal areas [168]. Overall, the current literature has suggested that the opioid system plays a major role in fat preference.

#### 3.2.4. Other Mechanisms that Potentially Influence Macronutrient Preference

Several studies have reported a link between genetics and macronutrient preferences. An investigation into the interactions between obesity-related copy number variants and dietary behaviors in childhood obesity revealed a prominent multiplicative interaction between genetic 10q11.22 deletions and the preference for a meat-based diet [169]. This chromosomal region contains 14 genes, including the *PPYR1* gene, which encodes the NPY4 receptor. The NPY4 receptor binds both orexigenic NPY and anorexigenic pancreatic polypeptide [170].

Other GWAS have linked *FGF21* and the fat mass and obesity-associated gene, *FTO*, to macronutrient-specific preferences [171,172,173]. Genetic variations in the *FTO* locus are associated with obesity [174]. The rs9939609 obesity-risk allele of the *FTO* gene was reported to be associated with sucrose and protein preferences [175], high-fat and low fiber intakes [176], and carbohydrate and protein intakes [177]. FTO is highly expressed in brain regions that control feeding and energy expenditure, including the hypothalamus [178]. *FTO* encodes a nucleic acid demethylase, which demethylates N^6^-methyladenosine (m^6^A) and N^6^,2′-*O*-dimethyladenosine (m^6^Am) on RNA. This demethylation activity controls mRNA stability [178,179,180]. One study in mice identified neurons that expressed both FTO and Oxt, and FTO overexpression in a murine hypothalamic cell line increased Oxt mRNA levels [181]. Another study reported that FTO overexpression reduced m^6^A methylation on ghrelin mRNA and increased the levels of ghrelin mRNA and peptide in cell culture models [182]. The FTO genotype was reported to modulate neural responses to food images in homeostatic and brain reward regions [182]. Therefore, FTO may modulate macronutrient preference by acting on multiple targets.

The gut microbiome was reported to affect multiple aspects of health, including obesity [183]. Recently, a reduced abundance of *Bacteroides thetaiotaomicron* was observed in obese individuals. In mice, a gavage of the *B. thetaiotaomicron* was reported to alleviate diet-induced obesity [184]. In another study, a maternal HFD was reported to cause dysbiosis and to alter the gut microbiome of offspring. In particular, *Lactobacillus reuteri* was reduced, and the social behavior of offspring was affected, due to reduced PVN Oxt levels [185]. Importantly, reconstituting the offspring’s gut microbiota by supplementing the gut with *L*. *reuteri* restored social behavior and Oxt levels [185]. These data suggested that gut microbiota, such as *L. reuteri*, may modulate carbohydrate preference through Oxt expression; however, this hypothesis requires testing.

### 3.3. Concluding Remarks on Macronutrient Preference

It is evident that macronutrient-based diet selection is important for maintaining weight and health. Several molecules and systems are implicated in the regulation of macronutrient preference; however, they comprise fragmented knowledge on the neural and molecular mechanisms that control macronutrient preference. We currently lack an integrated understanding that connects all the fragmented information. Some molecular systems may imply the participation of a particular signaling pathway, but the possibilities must be tested. Further research would contribute to a more complete picture of the mechanisms that regulate macronutrient preference.

Once we attain an integrated view of the physiological mechanisms that control macronutrient preference, we can begin to apply that knowledge to investigate hidden pathologies that drive altered feeding behaviors in patients affected by obesity and diabetes. A better understanding of the physiology and pathophysiology of macronutrient-based diet selection behaviors is likely to lead to the development of better measures to facilitate implementation of macronutrient-balanced diets.

## 4. Feeding Pattern (When We Eat)

Feeding patterns in humans are influenced by internal appetite and the external environment. We eat when we are hungry, we eat three meals a day by custom, and we eat additional amounts at social occasions. We may also skip a meal, due to, for example, time constraints or work shifts. Observations in animals under *ad libitum* experimental conditions have shed light on the rhythmic nature of the internal appetite and mechanisms that generate the feeding pattern. The results have generated additional questions, like how important is the synchronization between the internal appetite and the actual ingestion of food, and how is the feeding pattern generated?

### 4.1. Importance of Feeding Patterns for Controlling Weight and Health

When we eat is another factor that influences health. “Chrono-nutrition” refers to coordinating the time of food ingestion with the body’s daily rhythm [186]. Ingesting the same food at different times of the day has different consequences on health, because systemic metabolic efficiency fluctuates over the course of the day [187]. In humans, de-synchronization between feeding time and the circadian clock was imposed by simulating night-shift work. This condition reduced energy expenditure [188] and led to hyperglycemia and insulin resistance [189]. The duration of a night-shift work rotation was associated with greater risks of obesity and type 2 diabetes [190]. A five-year prospective, longitudinal study identified that, among adolescents, breakfast consumption was inversely associated with weight gain [191]. Within a cohort of patients that participated in a behavioral weight-loss program, those who ingested more calories earlier in the day were more likely to lose weight [192]. During an isocaloric weight-loss program, better improvements in metabolic markers were achieved in the group that had a bigger breakfast and a smaller dinner, compared to those that ate later [193]. Scheduling three meals earlier, significantly improved serum lipid levels [194], and conversely, night-eating syndrome, characterized by a delayed meal pattern, was positively associated with body mass index (BMI) [195]. Snacking independent of meals contributed to hepatic steatosis and obesity in humans [196]. Therefore, meal timing clearly affects health in humans.

Disruptions in biological rhythms are linked to obesity and associated metabolic consequences [197]. In mice, a genetic disruption of the circadian clock promoted obesity and leptin resistance and disturbed glucose and lipid homeostasis [197,198,199]. In Zucker rats, obesity progression was associated with disturbances in the feeding rhythm [200], and improper timing of food intake was linked to weight gain in both rodents [201] and humans [202]. Correcting the feeding schedule was sufficient to prevent HFD-induced obesity in mice, even when the amount of calories ingested was unaltered [203,204,205]. Food intake during the normal activity phase prevented obesity and circadian desynchrony in a rat model of night work [206]. In nocturnal mice, early nocturnal fasting increased body weight, because it promoted compensatory feeding during other times of the day, and late nocturnal fasting reduced weight gain [207,208]. Ingesting food during an early dark cycle is important for rodents, presumably because mice can use fat more efficiently during the earlier phase of the dark cycle than during the later dark phase and the light phase [187]. Collectively, evidence has emphasized the importance of feeding rhythms in maintaining energy balance and health across multiple species.

The next issue is how *ad libitum* feeding patterns are regulated. To tackle this issue, one must first understand the circadian regulation of rhythmic behaviors.

### 4.2. Molecular and Neural Bases for Circadian Regulation of Various Rhythms

To understand how the feeding pattern is generated, one must first understand the circadian clock system that regulates biological rhythms. Various biological rhythms are regulated by a master clock, located in the suprachiasmatic nucleus (SCN) of the hypothalamus [197]. Although the SCN is considered the main biological clock, several brain areas and organs are also known as brain clocks and peripheral clocks, respectively [209]. Both the master clock and the peripheral clocks use a molecular clock mechanism driven by a transcription-translation feedback loop [210]. The main feedback loop consists of the Bmal1/Clock transcription factor complex, which induces the expression of the *Per* (Period) and *Cry* (Cryptochrome) genes. The Per/Cry protein complex then inhibits the activity of the Bmal1/Clock complex [211].

The SCN is primarily entrained by the light/dark cycle, via a monosynaptic pathway called the retinohypothalamic tract. This tract projects from photosensitive melanopsin-containing retinal ganglion cells in the inner retina to the SCN [212,213,214,215,216,217,218]. The SCN also receives neural afferents from the median raphe serotonergic pathway and from the NPY-containing thalamic intergeniculate leaflet (geniculohypothalamic tract) [219]. The SCN sends direct and indirect projections to the VTA, through the lateral habenula, through the medial preoptic nucleus of the hypothalamus, and through orexin neurons located in the LH via the DMH [220,221,222,223,224]. The SCN also sends monosynaptic and polysynaptic projections to the ARC [225], PVN [226], LH [227], and DMH [225]. Efferent projections from the SCN are principally GABAergic, and they serve to repress neuronal activity outside the SCN [225].

In contrast, some peripheral clocks are primarily influenced by feeding [228]. Among the peripheral tissues, liver and adipose tissues are entrained by nutrients, food intake, and meal timing [228,229,230]. Because the primary zeitgebers (environmental timing signal that modulates the internal clock) of the SCN are different from those of the peripheral clocks, the rhythms generated by oscillators in peripheral tissues must be synchronized by the central SCN clock to ensure that systemic physiologies are in synchrony.

The SCN generates a near 24-h rhythmic output, and regulates slave clocks located within the brain and the periphery, to coordinate various rhythms systemically. The SCN synchronizes subordinate organ and tissue clocks with electrical, endocrine, and metabolic signaling pathways, namely, neural outputs and humoral mediators [209]. The SCN also controls the balance of sympathetic and parasympathetic activity transmitted to peripheral organs [231]. Several humoral mediators have been postulated to mediate the synchronization of peripheral clocks by the SCN and the synchronization of different neurons within the SCN, such as prokineticin 2 [232,233,234], arginine vasopressin [235,236], cardiolipin-like cytokine [237], vasoactive intestinal polypeptide [238], orexin [239,240,241], pituitary adenylate cyclase-activating peptide [242,243,244], transforming growth factor-alpha [245], melatonin [246,247], and corticosterone [248,249]. However, the complete array of mechanisms that the SCN master clock uses to control internal synchronization of slave clocks remains to be elucidated [211].

### 4.3. Possible Mechanisms that Regulate Feeding Patterns

It has been proposed that individual brain clocks exist for controlling the rhythms of feeding, activity, metabolism, and the sleep/wake cycle. However, the precise location of the “feeding-specific clock” in the brain remains an issue of debate.

#### 4.3.1. Food Anticipatory Activity (FAA) and the Food-Entrained Oscillator (FEO)

Food anticipatory activity (FAA) is a food-seeking behavior that operates when the chance of obtaining food is high. FAA is likely to be an adaptive strategy to enhance foraging success [250]. Restricted daily feeding induces oscillations from the SCN-independent food-entrained oscillator (FEO). The FEO operates even in the brains of SCN-lesioned rats [251,252]. FEO control of FAA has been studied extensively with time-restricted feeding paradigms, but its precise molecular and circuit-level identities have remained elusive. Candidate locations of brain structures that might harbor the FEO are the cerebellum [253], the DMH [254,255,256], the dorsal striatal circuits [257], the mesocorticolimbic circuits [258,259], and the VMH [260]. Dopamine [257], orexin [240], and melanocortin type 3 receptors [261] have been proposed to participate in FEO. The DMH has been proposed to participate in FAA, but its role remains controversial [262]. The VMH was reported to relay nutritional inputs and regulate activity behavior and energy expenditure [263,264]. Circadian behavior in the VMH is connected to food intake through SIRT1 [263], a metabolic sensor important for body weight regulation in the ARC [1,265,266]. *Sirt1* knockout mice exhibited a marked reduction in FAA [267,268].

When mice were placed under constant darkness conditions, timed delivery of palatable food, in addition to *ad libitum* regular chow feeding, entrained a behavioral rhythm [269]. This finding suggested that the reward system was involved in this entrainment. However, studies have reported conflicting findings, which suggested that either dopamine D1 [257] or D2 receptors [270] were important for FAA. The importance of molecular clock machinery in FAA is also controversial. CNS-specific *Bmal1* knockout mice showed delayed and attenuated FAA [271]; moreover, *Per1/Per2/Per3* triple knockout mice showed unstable, imprecise FAA [272]. However, FAA persisted in mice with a mutated *clock* gene [273] and in mice with null expression of *Npas2* [274], *Cry1/Cry2* [275,276], *Bmal1* [277,278], *Per1*, *Per2*, and *Per1/Per2* [278].

The FEO may be driven by multiple oscillators. The experimental approach of eliminating the function of one oscillator may not effectively reveal the entire mechanism, due to compensation by other oscillators within the system.

**Figure 2:**
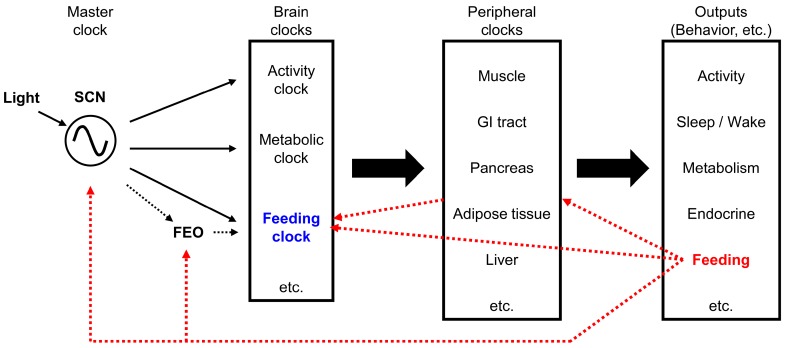
Biological clock systems and feeding behavior. Multiple layers of biological clocks regulate biological outputs. Red arrows indicate how feeding behavior can send feedback to the feeding clock, by affecting multiple levels within the system. Abbreviations: FEO, food-entrained oscillator; SCN, suprachiasmatic nucleus of the hypothalamus.

Concepts regarding biological clocks and feeding signals are summarized in Figure 2.

#### 4.3.2. Are the FEO and the *Ad Libitum* Feeding Pattern Generator Identical

Conceptually, the FEO and the endogenous *ad libitum* feeding pattern generator (feeding clock) are not identical. Studies that investigate the FEO are focused on how the internal clock is modulated by feeding. Feeding is considered an external zeitgeber, and most studies address how feeding regulates the FEO, not vice versa. In contrast, investigations on the mechanisms that regulate the *ad libitum* feeding pattern focus on identifying the feeding clock that generates the feeding pattern. Those studies consider feeding an output that reflects the rhythm of the feeding clock. Although feeding/fasting cycles are mainly driven by the SCN, through rest-active cycles, feeding patterns can be influenced by other factors. Therefore, the *ad libitum* feeding pattern generator and FEO are not likely to be identical.

The time scales observed in studies that investigate these two phenomena are also different. FAA, induced by time-restricted feeding, takes several days to develop [279]; moreover, FAA induced by palatable food access is relatively weak, and it tends to develop slowly over the course of several weeks [280]. In contrast, a disruption of the endogenous *ad libitum* feeding pattern occurs within a day of initiating HFD feeding [281].

These data indicate that the time scales of observed phenotypes and the causal directionality toward feeding are different between the FEO and the endogenous *ad libitum* feeding pattern. Consequently, to elucidate the mechanisms that regulate the endogenous *ad libitum* feeding pattern, it is necessary to find a different approach from those used to study the FEO.

#### 4.3.3. Neurotransmitter Systems That Show Diurnal Rhythms

Several neurotransmitter systems show diurnal variations. This information may provide clues for identifying the feeding clock that generates the *ad libitum* feeding pattern.

The dopaminergic system shows diurnal variations [282]. The expression and protein levels of tyrosine hydroxylase (TH) in the VTA show diurnal rhythms [282,283,284]. In addition, extracellular dopamine concentrations in the striatum vary in a 24-h cycle [285,286,287,288]. In rats, extracellular concentrations of dopamine in the NAc, mPFC, and dorsal striatum showed diurnal rhythmicity; concentrations were high during the active period, when rats were housed under constant dark conditions [286,288,289,290,291]. Dopamine removal from the synapse also showed a diurnal rhythm—in rodents, the expression and activity of a dopamine degrading enzyme, monoamine oxidase A, and dopamine clearance, showed peaks in the mid-to-late day, in the NAc and dorsal striatum [288,292,293,294]. In addition, the influence of orexinergic inputs on VTA dopamine activity fluctuated diurnally, with greater activation during the dark phase [221].

Potential links have been proposed between the SCN and the dopamine system [295]. Electrolytic lesions of the SCN clock altered the diurnal changes in TH and dopamine transporter protein levels, in the NAc and the striatum. Those findings suggested that dopamine activity in the forebrain and midbrain depend on the SCN [294]. Therefore, ample evidence has indicated that the activity of the dopamine system shows diurnal rhythms, and the SCN may influence the rhythmicity. Conversely, the dopamine system may also influence the SCN. In rats, a knock-down of the circadian gene, *Clock*, in the VTA shortened the free-running period of the wheel-running rhythm. This result indicated that the VTA rhythm affected SCN function [296]. Dopamine receptors are expressed in the SCN in rodents [297] and macaques [298]. D1-receptor-positive neurons in the SCN are important, because they are the dominant pace-making elements in the SCN circuit [211,299]. Methamphetamine, a strong stimulant of the dopaminergic and noradrenergic systems, affected daily rhythms in animals exposed to light-dark cycles or constant darkness [300]. The methamphetamine-sensitive circadian oscillator (MASCO) could restore circadian rhythms of behavior and other parameters, when methamphetamine was given *ad libitum* in drinking water [252]. Those results indicated that an SCN-independent circadian oscillator was present. Methamphetamine can change the resetting responses to brief light stimulation and the entrainment to a light-dark cycle [252,301]. These findings suggested that the dopamine system and MASCO may modulate the rhythmicity of the SCN.

In rats, the expression levels of NPY and its receptor, Y1R, in the ARC and PVN are higher during the active period [302]. NPY synthesis and release in the rat hypothalamus showed diurnal rhythmicity; they peaked prior to the nocturnal feeding period, under *ad libitum* feeding conditions, and prior to the scheduled daily meal-time, under time-restricted feeding conditions [303,304,305]. The ablation of NPY signaling in the mediobasal hypothalamus (MBH) of rats increased feeding during the light period [306]. However, *Npy*-knockout mice showed FAA by day 14, although FAA was reduced on day 7 of time-restricted daytime feeding [307]. Ghrelin-responsive ventromedial ARC neurons, which are likely to express both NPY and AgRP, communicate peripheral metabolic information to the SCN [308]. The SCN can also sense NPY input from the thalamic intergeniculate leaflet [309,310]. Ablation of leptin-sensitive neurons in the ARC did not disrupt the nocturnal feeding pattern under a proper light-dark cycle, but the feeding pattern became arrhythmic under continuous light and constant darkness conditions. These results suggested that ARC neurons are important for maintaining a circadian *ad libitum* feeding pattern, independent of light entrainment [311]. The overexpression of NPY in the LH, but not in the PVN, reduced the amplitude of locomotor activity and disrupted the diurnal eating pattern [312].

The neuronal histamine system showed diurnal variations: histaminergic neurons were active during the wake cycle (night-time in rodents, day-time in monkeys and humans) and depressed during the sleep cycle [313]. Histaminergic fibers from the tuberomamillary nucleus in the posterior hypothalamus project to the SCN and several other regions known to be linked to the SCN [313]. Selective deletion of *Bmal1* in histaminergic neurons of the posterior hypothalamus affected behavioral activity rhythms and caused fragmented sleep [314]. Hypothalamic neuronal histamine regulated feeding through histamine H1 receptors, and mice with a genetic knockout of the H1 receptor showed impaired diurnal feeding rhythms [315]. However, the precise location of the neurons that express the H1 receptor responsible for this regulation, remains unknown.

### 4.4. HFD and Feeding Patterns

HFDs have a significant impact on feeding behavior. HFDs affect both the intake volume and the feeding pattern, and they disrupt various rhythms. Here, we describe the effects of HFDs on various rhythms, on the hypothalamus, and on feeding patterns.

#### 4.4.1. Impact of HFDs on Various Rhythms

HFDs promote obesity by causing excessive energy intake and by disturbing various rhythms, such as feeding, locomotor activity, and metabolism [281,316,317]. Nevertheless, the main behavioral output most influenced by HFD is feeding behavior. In rodents, HFDs alter feeding patterns; they change from eating consolidated large meals, under normal chow feeding conditions, to small, but frequent meals, which increase in number and may extend into the resting period, under HFD conditions [281,316,318,319]. These changes in feeding patterns result in the desynchronization of energy intake and expenditure [320].

In nocturnal rodents fed normal chow, locomotor activity is highest during the early dark cycle, but some bouts of activity are also observed during the light cycle. However, HFD feeding disrupts this activity pattern in rodents. HFD decreases the amount of activity observed during the dark cycle [203,281,317], and disrupts activity bouts during the light cycle. The result is arrhythmic activity [281,318]. HFD feeding also disrupts sleep-wake physiology in rodents; it reduces waking times, it causes wake fragmentation, and it alters rapid-eye-movement (REM) and non-REM sleep patterns [321,322,323].

#### 4.4.2. Impact of HFD on Feeding Regulatory Mechanisms

HFDs induce hyperphagia, partly by causing hypothalamic inflammation. Hypothalamic inflammation was induced within 1 to 3 days of the onset of HFD feeding, prior to substantial weight gain [324]. HFD feeding caused hypothalamic inflammation restricted to the ARC; no inflammation was observed in the adjacent VMH or other hypothalamic areas [324]. HFD feeding caused hypothalamic accumulation of proinflammatory lipids, such as long-chain saturated fatty acids (SFAs) [325,326]. Among the SFAs, palmitic acid (C16:0) and stearic acid (C18:0) in particular, caused significant inflammation-induced activation of microglia [327]. These long-chain SFAs predominantly activated toll-like receptor 4 (TLR4) signaling in the hypothalamus [328], which triggered nuclear factor kappa B (NF-κB) activation and led to the expression of proinflammatory genes in the hypothalamus [325,328,329]. Although microglia and astrocytes accumulated in the MBH of mice fed HFDs, only the microglia underwent inflammatory activation when hypothalamic SFAs accumulated [327]. Pharmacological depletion of microglia or selective inhibition of microglial NF-κB signaling, sharply reduced hypothalamic microgliosis caused by HFD feeding [330]. Forcing microglial activation by cell-specific NF-κB signaling activation induced spontaneous MBH microgliosis and enhanced myeloid cell recruitment into the MBH [330]. Peripheral myeloid cells, in conjunction with astrocyte inflammatory signaling, enhanced established diet-induced obesity and hypothalamic inflammation [330,331]. The resulting hypothalamic inflammation caused resistance to signaling by-hormones, such as leptin and insulin, and disrupted energy homeostasis [1,332,333].

HFD feeding also affected the reward system. HFD feeding *ad libitum* reduced the reward value of food (and cocaine) in conditioned place preference paradigms [334]. Reorganizing the feeding pattern alleviated the reward deficit evoked by an HFD [334]. That finding suggested that disturbing the feeding pattern could affect the reward system. Therefore, HFD feeding can affect homeostatic feeding and hedonic feeding, in addition to changing the circulating humoral factors that reach these centers.

#### 4.4.3. HFD Disruption of Feeding Patterns

HFDs can affect feeding patterns through several potential targets. Here, we describe four potential targets:

1. The SCN

One hypothesis is that the HFD affects the master clock in the SCN and disturbs various biological rhythms, including feeding, activity, and sleep/wake patterns. HFDs can raise the level of SFAs in the hypothalamus [326], which might also affect the molecular clock, because the components of the clock are responsive to circulating nutrients, feeding-related hormones, and cellular redox and energy states [260]. In diet-induced obese mice, the expression of *Bmal1* (brain and muscle ARNT-like 1), one of the clock genes, was reduced in the SCN under constant dark conditions [335]; in those conditions, light could not serve as an external zeitgeber to the SCN to adjust its circadian rhythmicity. HFD feeding increased the period of the circadian pacemaker, and it advanced the phase of the liver clock [230,318]; therefore, HFD feeding can promote a dissociation between the circadian rhythmicities of the central pacemaker and peripheral clocks, such as the liver,.

However, a previous study showed that a 12-week HFD feeding period had no effect on the molecular oscillations in the SCN, ARC, or DMH [336]. Therefore, it is unlikely that the instant disturbance in feeding rhythms, induced by a HFD, results from a direct effect on the molecular clock within the SCN.

2. The Dopamine System

Because the HFD is rewarding, switching from normal chow to a HFD at the beginning of the light cycle promptly increases the intake of the HFD within the first light cycle and disrupts the feeding pattern. One could argue that, for nocturnal rodents, eating during the dark cycle is homeostatic, but not eating during the light cycle. Non-homeostatic light cycle feeding might be driven by the hedonic value of a HFD, which would suggest that the mesolimbic dopamine system might be involved. It has been proposed that potential reciprocal links exist between the SCN and the dopamine system [295]. However, it remains to be explained, both molecularly and histologically, how HFD feeding affects the mesolimbic dopamine system acutely within the first several hours after a diet switch.

3. ARC NPY/AgRP Neurons

Within a day of initiation, HFD feeding causes acute microglia-mediated ARC inflammation [324]. The ARC is the primary center of feeding control. ARC neurons communicate with the SCN, and the SCN can sense NPY [308,309,310]. ARC neurons are important for maintaining a circadian *ad libitum* feeding pattern, independent of light entrainment [311]. Thus, an inflammation-perturbed ARC may be responsible for the aberrant feeding pattern evoked by HFD feeding.

4. Nutrient Information Entering the Brain

Another hypothesis is that SFAs affect the peripheral clocks that feed back to the central clock and/or the communication between two clocks [337,338], and thus, they indirectly disturb the central clock. For instance, HFD feeding disturbs circadian gene expression profiles in the liver [230]. HFDs also ablate the gastric vagal afferent circadian rhythm. HFD feeding for 12 weeks resulted in the loss of circadian fluctuations in stomach content, consistent with a disrupted feeding pattern in mice [338]. Mice fed HFDs exhibited a loss of circadian fluctuations in gastric vagal afferent mechanosensitivity, which are observed in mice fed regular chow [338].

HFD feeding can also affect humoral signaling. In addition to altering circulating humoral profiles, HFD feeding also blunts the access of humoral factors to target neurons by inducing signaling resistance at multiple levels [1]. The combined effects on input signals to the brain and the sensitivity of the brain to the input signals could induce both the feeding system and the circadian system to adjust to the new environment with different outputs, which could lead to altered feeding behavior.

**Figure 3:**
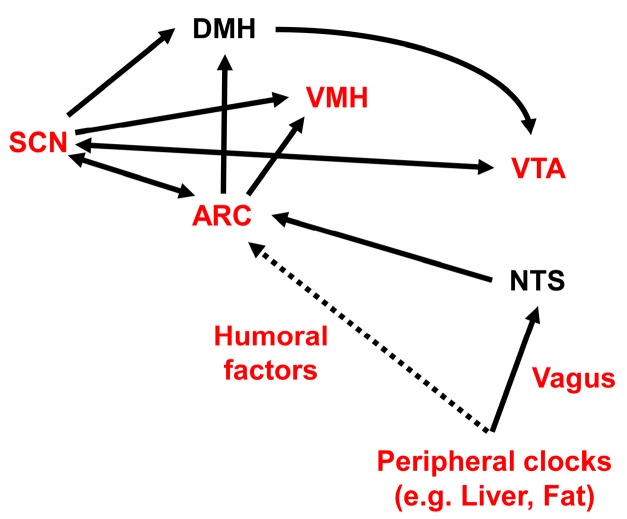
The high fat diet (HFD) affects neuronal nodes that are important for feeding and for the circadian system. Nodes and pathways shown in red have been reported to be affected by HFD feeding. Abbreviations: ARC, arcuate nucleus of the hypothalamus; DMH, dorsomedial nucleus of the hypothalamus; NTS, nucleus of the solitary tract; SCN, suprachiasmatic nucleus of the hypothalamus; VMH, ventromedial nucleus of the hypothalamus; VTA, ventral tegmental area.

Therefore, it remains to be determined how the feeding rhythm is regulated physiologically and how it is disturbed by HFDs. The HFD may disturb feeding patterns by affecting multiple layers of clock and feeding machineries (Figure 3).

### 4.5. Concluding Remarks on Feeding Patterns

Compared to the mechanisms underlying macronutrient preferences, the mechanisms that regulate endogenous *ad libitum* feeding patterns remain mostly unidentified. Consequently, it is difficult to speculate about the interactions and relationships that might occur among known components. More clues are required. Research that focuses on endogenous *ad libitum* feeding rather than feeding as a zeitgeber, could facilitate the identification of novel molecules and neural nodes that contribute to the neural circuits that generate and regulate endogenous *ad libitum* feeding patterns.

Feeding patterns observed in experimental animals represent endogenous rhythms in appetite. Although eating patterns in humans are influenced by both the endogenous appetite and external environmental cues, a dissociation between these two influences could cause psychological stress and promote unnecessary snacking. Therefore, research on endogenous feeding patterns may reveal ways to alleviate this stressful dissociation and lead to healthier eating patterns.

## 5. Conclusions

Feeding affects biology through three parameters: what, when, and how much. Studies that focus on “how much” we eat have paved the way to a better understanding of neural and molecular mechanisms that control feeding behavior. The remaining factors, “what” and “when” we eat, are equally important for health, but the central mechanisms that regulate these processes are less well understood. What we eat influences when we eat and affects how much we eat. The presence of food choices influences meal patterns and total caloric intake, even in mice [339,340].

However, many open questions remain unaddressed in the field. Here, I provide a list of open questions for each topic to stimulate future investigations.

Open questions regarding the regulatory mechanisms that regulate macronutrient preference:

(1-1) How is macronutrient intake sensed by the neural circuitry?
  (1-1-1) Could dietary components (like glucose) and intermediate metabolites directly affect neural nodes that regulated feeding behavior?
  (1-1-2) Are there unidentified bioactive molecules/hormones that mediate the transmission of nutrient information from the peripheral tissues to the central nervous system?
  (1-1-3) Does a mechanism exist that can sense the lengths of fatty acid chains (e.g., short-chain vs. long-chain), and thus, regulate preferences for specific fatty acids? For example, FGF21 assumes this role in the context of carbohydrates, and it regulates simple sugar-specific preferences (as discussed in the Section 3.2.1).
(1-2) Where and how is feeding-related multimodal sensory information integrated within the brain? We may need better model organisms to study such processes, because rodent models cannnot recapitulate all the features of sensory processing observed in humans.
(1-3) How do homeostatic, hedonic, and possibly multimodal integration system(s) interact with each other? The homeostatic and hedonic systems are known to interact at multiple neural nodes, but our current knowleadge is likely to be incomplete.

Open questions regarding the feeding patterns:

(2-1) What is the identity of the feeding pattern generator?
  (2-1-1) Is it located in the brain or in the peripheral tissues?
  (2-1-2) Is it a single master regulator, or is it influenced by multiple oscillators?
(2-2) Despite the fact that molecular clock system that generates circadian rhythms is conserved among multiple species, nocturnal and diurnal organisms eat at different times of the day. Therefore, are the mechanisms that regulate feeding patterns conserved among multiple species, particularly nocturnal and diurnal organisms?
  (2-2-1) If the mechanisms are conserved, do different species have different way(s) of coordinating the molecular clock with the feeding pattern generator(s)?
  (2-2-2) If the mechanisms are not conserved, what animal models might be best for investigating the neural mechanisms responsible for generating different feeding patterns?
(2-3) To what extent does the *ad libitum* feeding pattern generator dictate the feeding behavior in humans?

Open questions regarding the importance of these processes in the context of obesity and weight control:

(3-1) How are the mechanisms that regulate macronutrient selection and feeding patterns regulated or altered in obesity?
(3-2) If some of the mechanisms are altered, are these changes reversible upon weight loss?
(3-3) If reversible, are they always reversible, or is there a point of no return?

This review has shown that it is important to study the mechanisms that regulate the quality of feeding. Addressing the above questions and understanding the underlying mechanisms can lead to identifying what goes wrong in pathology. Identifying the pathological mechanisms involved in both the quantity and quality of feeding will eventually lead to the development of better dietary interventions for preventing weight gain, promoting weight loss, and resisting weight regain.

## Figures and Tables

**Table 1:** Simplified comparison of “wanting” vs. “liking”.

“Wanting”	Appetitive (Before Ingestion)	“Want to Eat!”	Motivation Incentive Salience	Dopamine System
“Liking”	Consummatory (after ingestion)	“Delicious!”	Pleasure Hedonic impact	Opioid system

**Table 2:** Central melanocortin system mutants and macronutrient preference.

	*MC4R* Mutation (Human)	*Pomc*-Null (Mice)	*Ay/a* (Mice)
Fat preference	Increase	Increase	Increase
Carbohydrate preference	Decrease	N.D. ^1^	Decrease

^1^ Not determined.

**Table 3:** The relationships between neuropeptides and macronutrients.

Macronutrient	Galanin	NMU	NPY	Oxt	MCH	CRH
Neuropeptide effect on fat preference	Increase (PVN)	Decrease (PVN)	Increase (NAc)	(-)	N.D. ^1^	Increase (-)
Effect of fat ingestion on peptidergic function	Increase (PVN)	N.D. ^1^	N.D. ^1^	N.D. ^1^	N.D. ^1^	N.D. ^1^
Neuropeptide effect on carbohydrate preference	(-)	(-)	Increase (PVN, ICV)	Decrease	Increase vs. (-)	Increase vs. decrease
Effect of carbohydrate ingestion on peptidergic function	Decrease (PVN)	N.D. ^1^	Increase (ARC, PVN)	Increase (PVN)	N.D. ^1^	N.D. ^1^

^1^ Not determined; (-): no effect. Abbreviations: CRH, corticotropin releasing hormone; ICV: intracerebroventricular injection; MCH, melanocyte concentrating hormone; NAc, nucleus accumbens; NMU, neuromedin U; NPY, neuropeptide Y; Oxt, oxytocin; PVN, paraventricular nucleus of the hypothalamus.

## References

[1] Sasaki T. (2015). Age-associated weight gain, leptin, and sirt1: A possible role for hypothalamic sirt1 in the prevention of weight gain and aging through modulation of leptin sensitivity. Front. Endocrinol. (Lausanne).

[2] GBD2015 Risk Factors Collaborators (2016). Global, regional, and national comparative risk assessment of 79 behavioural, environmental and occupational, and metabolic risks or clusters of risks, 1990–2015: A systematic analysis for the global burden of disease study 2015. Lancet.

[3] McDaid A.F., Joshi P.K., Porcu E., Komljenovic A., Li H., Sorrentino V., Litovchenko M., Bevers R.P.J., Rueger S., Reymond A. (2017). Bayesian association scan reveals loci associated with human lifespan and linked biomarkers. Nat. Commun..

[4] GBD2015 DALYs and HALE Collaborators (2016). Global, regional, and national disability-adjusted life-years (dalys) for 315 diseases and injuries and healthy life expectancy (hale), 1990–2015: A systematic analysis for the global burden of disease study 2015. Lancet.

[5] Gletsu-Miller N., McCrory M.A. (2014). Modifying eating behavior: Novel approaches for reducing body weight, preventing weight regain, and reducing chronic disease risk. Adv. Nutr..

[6] Soeliman F.A., Azadbakht L. (2014). Weight loss maintenance: A review on dietary related strategies. J. Res. Med. Sci..

[7] Molin Netto B.D., Earthman C.P., Farias G., Landi Masquio D.C., Grotti Clemente A.P., Peixoto P., Bettini S.C., von Der Heyde M.E., Damaso A.R. (2017). Eating patterns and food choice as determinant of weight loss and improvement of metabolic profile after rygb. Nutrition.

[8] Drewnowski A., Holden-Wiltse J. (1992). Taste responses and food preferences in obese women: Effects of weight cycling. Int. J. Obes. Relat. Metab. Disord..

[9] Reed D.R., Contreras R.J., Maggio C., Greenwood M.R., Rodin J. (1988). Weight cycling in female rats increases dietary fat selection and adiposity. Physiol. Behav..

[10] Sasaki T., Matsui S., Kitamura T. (2016). Control of appetite and food preference by nmda receptor and its co-agonist d-serine. Int. J. Mol. Sci..

[11] Berthoud H.R., Morrison C. (2008). The brain, appetite, and obesity. Annu. Rev. Psychol..

[12] Basiri M.L., Stuber G.D. (2016). Multimodal signal integration for feeding control. Cell.

[13] Andermann M.L., Lowell B.B. (2017). Toward a wiring diagram understanding of appetite control. Neuron.

[14] Brookes S.J., Spencer N.J., Costa M., Zagorodnyuk V.P. (2013). Extrinsic primary afferent signalling in the gut. Nat. Rev. Gastroenterol. Hepatol..

[15] Friedman J.M., Halaas J.L. (1998). Leptin and the regulation of body weight in mammals. Nature.

[16] Wada N., Hirako S., Takenoya F., Kageyama H., Okabe M., Shioda S. (2014). Leptin and its receptors. J. Chem. Neuroanat..

[17] Lee G.H., Proenca R., Montez J.M., Carroll K.M., Darvishzadeh J.G., Lee J.I., Friedman J.M. (1996). Abnormal splicing of the leptin receptor in diabetic mice. Nature.

[18] Chen H., Charlat O., Tartaglia L.A., Woolf E.A., Weng X., Ellis S.J., Lakey N.D., Culpepper J., Moore K.J., Breitbart R.E. (1996). Evidence that the diabetes gene encodes the leptin receptor: Identification of a mutation in the leptin receptor gene in db/db mice. Cell.

[19] Havrankova J., Roth J., Brownstein M. (1978). Insulin receptors are widely distributed in the central nervous system of the rat. Nature.

[20] Vogt M.C., Bruning J.C. (2013). Cns insulin signaling in the control of energy homeostasis and glucose metabolism—From embryo to old age. Trends Endocrinol. Metab..

[21] Kojima M., Hosoda H., Date Y., Nakazato M., Matsuo H., Kangawa K. (1999). Ghrelin is a growth-hormone-releasing acylated peptide from stomach. Nature.

[22] Cummings D.E., Purnell J.Q., Frayo R.S., Schmidova K., Wisse B.E., Weigle D.S. (2001). A preprandial rise in plasma ghrelin levels suggests a role in meal initiation in humans. Diabetes.

[23] Date Y., Murakami N., Toshinai K., Matsukura S., Niijima A., Matsuo H., Kangawa K., Nakazato M. (2002). The role of the gastric afferent vagal nerve in ghrelin-induced feeding and growth hormone secretion in rats. Gastroenterology.

[24] Le Roux C.W., Neary N.M., Halsey T.J., Small C.J., Martinez-Isla A.M., Ghatei M.A., Theodorou N.A., Bloom S.R. (2005). Ghrelin does not stimulate food intake in patients with surgical procedures involving vagotomy. J. Clin. Endocrinol. Metab..

[25] Date Y., Shimbara T., Koda S., Toshinai K., Ida T., Murakami N., Miyazato M., Kokame K., Ishizuka Y., Ishida Y. (2006). Peripheral ghrelin transmits orexigenic signals through the noradrenergic pathway from the hindbrain to the hypothalamus. Cell Metab..

[26] Cowley M.A., Smith R.G., Diano S., Tschop M., Pronchuk N., Grove K.L., Strasburger C.J., Bidlingmaier M., Esterman M., Heiman M.L. (2003). The distribution and mechanism of action of ghrelin in the cns demonstrates a novel hypothalamic circuit regulating energy homeostasis. Neuron.

[27] Steinert R.E., Feinle-Bisset C., Asarian L., Horowitz M., Beglinger C., Geary N. (2017). Ghrelin, CCK, GLP-1, and PYY(3–36): Secretory controls and physiological roles in eating and glycemia in health, obesity, and after rygb. Physiol. Rev..

[28] Chen H.Y., Trumbauer M.E., Chen A.S., Weingarth D.T., Adams J.R., Frazier E.G., Shen Z., Marsh D.J., Feighner S.D., Guan X.M. (2004). Orexigenic action of peripheral ghrelin is mediated by neuropeptide y and agouti-related protein. Endocrinology.

[29] Mullier A., Bouret S.G., Prevot V., Dehouck B. (2010). Differential distribution of tight junction proteins suggests a role for tanycytes in blood-hypothalamus barrier regulation in the adult mouse brain. J. Comp. Neurol..

[30] Krashes M.J., Lowell B.B., Garfield A.S. (2016). Melanocortin-4 receptor-regulated energy homeostasis. Nat. Neurosci..

[31] Sasaki T., Kitamura T. (2010). Roles of foxo1 and sirt1 in the central regulation of food intake. Endocr. J..

[32] Girardet C., Butler A.A. (2014). Neural melanocortin receptors in obesity and related metabolic disorders. Biochim. Biophys. Acta.

[33] Mercer R.E., Chee M.J., Colmers W.F. (2011). The role of npy in hypothalamic mediated food intake. Front. Neuroendocrinol..

[34] Berridge K.C., Robinson T.E. (2003). Parsing reward. Trends Neurosci..

[35] Berridge K.C., Robinson T.E., Aldridge J.W. (2009). Dissecting components of reward: ‘liking’, ‘wanting’, and learning. Curr. Opin. Pharmacol..

[36] Salamone J.D., Correa M. (2012). The mysterious motivational functions of mesolimbic dopamine. Neuron.

[37] Palmiter R.D. (2007). Is dopamine a physiologically relevant mediator of feeding behavior?. Trends Neurosci..

[38] Clark J.J., Hollon N.G., Phillips P.E. (2012). Pavlovian valuation systems in learning and decision making. Curr. Opin. Neurobiol..

[39] Norgren R., Hajnal A., Mungarndee S.S. (2006). Gustatory reward and the nucleus accumbens. Physiol. Behav..

[40] Liu S., Globa A.K., Mills F., Naef L., Qiao M., Bamji S.X., Borgland S.L. (2016). Consumption of palatable food primes food approach behavior by rapidly increasing synaptic density in the vta. Proc. Natl. Acad. Sci. USA.

[41] Small D.M., Jones-Gotman M., Dagher A. (2003). Feeding-induced dopamine release in dorsal striatum correlates with meal pleasantness ratings in healthy human volunteers. Neuroimage.

[42] Volkow N.D., Wang G.J., Fowler J.S., Telang F. (2008). Overlapping neuronal circuits in addiction and obesity: Evidence of systems pathology. Philos. Trans. R Soc. Lond. B Biol. Sci..

[43] Steinbusch L., Labouebe G., Thorens B. (2015). Brain glucose sensing in homeostatic and hedonic regulation. Trends Endocrinol. Metab..

[44] Figlewicz D.P., Evans S.B., Murphy J., Hoen M., Baskin D.G. (2003). Expression of receptors for insulin and leptin in the ventral tegmental area/substantia nigra (vta/sn) of the rat. Brain Res..

[45] Leshan R.L., Opland D.M., Louis G.W., Leinninger G.M., Patterson C.M., Rhodes C.J., Munzberg H., Myers M.G. (2010). Ventral tegmental area leptin receptor neurons specifically project to and regulate cocaine- and amphetamine-regulated transcript neurons of the extended central amygdala. J. Neurosci..

[46] Alhadeff A.L., Rupprecht L.E., Hayes M.R. (2012). Glp-1 neurons in the nucleus of the solitary tract project directly to the ventral tegmental area and nucleus accumbens to control for food intake. Endocrinology.

[47] Abizaid A., Liu Z.W., Andrews Z.B., Shanabrough M., Borok E., Elsworth J.D., Roth R.H., Sleeman M.W., Picciotto M.R., Tschop M.H. (2006). Ghrelin modulates the activity and synaptic input organization of midbrain dopamine neurons while promoting appetite. J. Clin. Investig..

[48] Jerlhag E., Egecioglu E., Dickson S.L., Douhan A., Svensson L., Engel J.A. (2007). Ghrelin administration into tegmental areas stimulates locomotor activity and increases extracellular concentration of dopamine in the nucleus accumbens. Addict. Biol..

[49] Fadel J., Deutch A.Y. (2002). Anatomical substrates of orexin-dopamine interactions: Lateral hypothalamic projections to the ventral tegmental area. Neuroscience.

[50] Hommel J.D., Trinko R., Sears R.M., Georgescu D., Liu Z.W., Gao X.B., Thurmon J.J., Marinelli M., DiLeone R.J. (2006). Leptin receptor signaling in midbrain dopamine neurons regulates feeding. Neuron.

[51] Labouebe G., Liu S., Dias C., Zou H., Wong J.C., Karunakaran S., Clee S.M., Phillips A.G., Boutrel B., Borgland S.L. (2013). Insulin induces long-term depression of ventral tegmental area dopamine neurons via endocannabinoids. Nat. Neurosci..

[52] Mebel D.M., Wong J.C., Dong Y.J., Borgland S.L. (2012). Insulin in the ventral tegmental area reduces hedonic feeding and suppresses dopamine concentration via increased reuptake. Eur. J. Neurosci..

[53] Skibicka K.P., Hansson C., Alvarez-Crespo M., Friberg P.A., Dickson S.L. (2011). Ghrelin directly targets the ventral tegmental area to increase food motivation. Neuroscience.

[54] Borgland S.L., Chang S.J., Bowers M.S., Thompson J.L., Vittoz N., Floresco S.B., Chou J., Chen B.T., Bonci A. (2009). Orexin a/hypocretin-1 selectively promotes motivation for positive reinforcers. J. Neurosci..

[55] Patyal R., Woo E.Y., Borgland S.L. (2012). Local hypocretin-1 modulates terminal dopamine concentration in the nucleus accumbens shell. Front. Behav. Neurosci..

[56] Sorensen G., Wegener G., Hasselstrom J., Hansen T.V., Wortwein G., Fink-Jensen A., Woldbye D.P. (2009). Neuropeptide y infusion into the shell region of the rat nucleus accumbens increases extracellular levels of dopamine. Neuroreport.

[57] Simon J.J., Wetzel A., Sinno M.H., Skunde M., Bendszus M., Preissl H., Enck P., Herzog W., Friederich H.C. (2017). Integration of homeostatic signaling and food reward processing in the human brain. JCI Insight.

[58] Toll L., Bruchas M.R., Calo G., Cox B.M., Zaveri N.T. (2016). Nociceptin/orphanin fq receptor structure, signaling, ligands, functions, and interactions with opioid systems. Pharmacol. Rev..

[59] Nogueiras R., Romero-Pico A., Vazquez M.J., Novelle M.G., Lopez M., Dieguez C. (2012). The opioid system and food intake: Homeostatic and hedonic mechanisms. Obes. Facts.

[60] Akil H., Watson S.J., Young E., Lewis M.E., Khachaturian H., Walker J.M. (1984). Endogenous opioids: Biology and function. Annu. Rev. Neurosci..

[61] Appleyard S.M., Hayward M., Young J.I., Butler A.A., Cone R.D., Rubinstein M., Low M.J. (2003). A role for the endogenous opioid beta-endorphin in energy homeostasis. Endocrinology.

[62] Le Merrer J., Becker J.A., Befort K., Kieffer B.L. (2009). Reward processing by the opioid system in the brain. Physiol. Rev..

[63] Cooper S.J. (1983). Effects of opiate agonists and antagonists on fluid intake and saccharin choice in the rat. Neuropharmacology.

[64] Cooper S.J., Turkish S. (1989). Effects of naltrexone on food preference and concurrent behavioral responses in food-deprived rats. Pharmacol. Biochem. Behav..

[65] Evans K.R., Vaccarino F.J. (1990). Amphetamine- and morphine-induced feeding: Evidence for involvement of reward mechanisms. Neurosci. Biobehav. Rev..

[66] Lynch W.C. (1986). Opiate blockade inhibits saccharin intake and blocks normal preference acquisition. Pharmacol. Biochem. Behav..

[67] Drewnowski A., Krahn D.D., Demitrack M.A., Nairn K., Gosnell B.A. (1992). Taste responses and preferences for sweet high-fat foods: Evidence for opioid involvement. Physiol. Behav..

[68] Fantino M., Hosotte J., Apfelbaum M. (1986). An opioid antagonist, naltrexone, reduces preference for sucrose in humans. Am. J. Physiol..

[69] Yeomans M.R., Gray R.W. (1996). Selective effects of naltrexone on food pleasantness and intake. Physiol. Behav..

[70] Zhang M., Kelley A.E. (2000). Enhanced intake of high-fat food following striatal mu-opioid stimulation: Microinjection mapping and fos expression. Neuroscience.

[71] Katsuura Y., Taha S.A. (2010). Modulation of feeding and locomotion through mu and delta opioid receptor signaling in the nucleus accumbens. Neuropeptides.

[72] Katsuura Y., Heckmann J.A., Taha S.A. (2011). Mu-opioid receptor stimulation in the nucleus accumbens elevates fatty tastant intake by increasing palatability and suppressing satiety signals. Am. J. Physiol. Regul. Integr. Comp. Physiol..

[73] Castro D.C., Berridge K.C. (2014). Opioid hedonic hotspot in nucleus accumbens shell: Mu, delta, and kappa maps for enhancement of sweetness “Liking” And “Wanting”. J. Neurosci..

[74] Kelley A.E., Bless E.P., Swanson C.J. (1996). Investigation of the effects of opiate antagonists infused into the nucleus accumbens on feeding and sucrose drinking in rats. J. Pharmacol. Exp. Ther..

[75] Yeomans M.R., Blundell J.E., Leshem M. (2004). Palatability: Response to nutritional need or need-free stimulation of appetite?. Br. J. Nutr..

[76] Hayward M.D., Schaich-Borg A., Pintar J.E., Low M.J. (2006). Differential involvement of endogenous opioids in sucrose consumption and food reinforcement. Pharmacol. Biochem. Behav..

[77] Mahler S.V., Berridge K.C. (2009). Which cue to “Want?” Central amygdala opioid activation enhances and focuses incentive salience on a prepotent reward cue. J. Neurosci..

[78] Mendez I.A., Ostlund S.B., Maidment N.T., Murphy N.P. (2015). Involvement of endogenous enkephalins and beta-endorphin in feeding and diet-induced obesity. Neuropsychopharmacology.

[79] Berridge K.C., Kringelbach M.L. (2015). Pleasure systems in the brain. Neuron.

[80] Drewnowski A., Henderson S.A., Levine A., Hann C. (1999). Taste and food preferences as predictors of dietary practices in young women. Public Health Nutr..

[81] Duffy V.B., Hayes J.E., Sullivan B.S., Faghri P. (2009). Surveying food and beverage liking: A tool for epidemiological studies to connect chemosensation with health outcomes. Ann. N. Y. Acad. Sci..

[82] (2015). The 2015 Food & Health Survey: Consumer Attitudes toward Food Safety, Nutrition & Health.

[83] Ventura A.K., Worobey J. (2013). Early influences on the development of food preferences. Curr. Biol..

[84] Novakovic R., Cavelaars A., Geelen A., Nikolic M., Altaba I.I., Vinas B.R., Ngo J., Golsorkhi M., Medina M.W., Brzozowska A. (2014). Socio-economic determinants of micronutrient intake and status in europe: A systematic review. Public Health Nutr..

[85] Pirastu N., Robino A., Lanzara C., Athanasakis E., Esposito L., Tepper B.J., Gasparini P. (2012). Genetics of food preferences: A first view from silk road populations. J. Food Sci..

[86] Feeney E., O’Brien S., Scannell A., Markey A., Gibney E.R. (2011). Genetic variation in taste perception: Does it have a role in healthy eating?. Proc. Nutr. Soc..

[87] Pallister T., Sharafi M., Lachance G., Pirastu N., Mohney R.P., MacGregor A., Feskens E.J., Duffy V., Spector T.D., Menni C. (2015). Food preference patterns in a uk twin cohort. Twin Res. Hum. Genet..

[88] Groschl M., Knerr I., Topf H.G., Schmid P., Rascher W., Rauh M. (2003). Endocrine responses to the oral ingestion of a physiological dose of essential amino acids in humans. J. Endocrinol..

[89] Knerr I., Groschl M., Rascher W., Rauh M. (2003). Endocrine effects of food intake: Insulin, ghrelin, and leptin responses to a single bolus of essential amino acids in humans. Ann. Nutr. Metab..

[90] Markus R., Panhuysen G., Tuiten A., Koppeschaar H. (2000). Effects of food on cortisol and mood in vulnerable subjects under controllable and uncontrollable stress. Physiol. Behav..

[91] Lehnert H., Wurtman R.J. (1993). Amino acid control of neurotransmitter synthesis and release: Physiological and clinical implications. Psychother. Psychosom..

[92] Wurtman R.J., Fernstrom J.D. (1976). Control of brain neurotransmitter synthesis by precursor availability and nutritional state. Biochem. Pharmacol..

[93] Acworth I.N., During M.J., Wurtman R.J. (1988). Tyrosine: Effects on catecholamine release. Brain Res. Bull..

[94] Lehnert H., Reinstein D.K., Strowbridge B.W., Wurtman R.J. (1984). Neurochemical and behavioral consequences of acute, uncontrollable stress: Effects of dietary tyrosine. Brain Res..

[95] Kuwata H., Iwasaki M., Shimizu S., Minami K., Maeda H., Seino S., Nakada K., Nosaka C., Murotani K., Kurose T. (2016). Meal sequence and glucose excursion, gastric emptying and incretin secretion in type 2 diabetes: A randomised, controlled crossover, exploratory trial. Diabetologia.

[96] Hall K.D., Bemis T., Brychta R., Chen K.Y., Courville A., Crayner E.J., Goodwin S., Guo J., Howard L., Knuth N.D. (2015). Calorie for calorie, dietary fat restriction results in more body fat loss than carbohydrate restriction in people with obesity. Cell Metab..

[97] Hall K.D., Guo J. (2017). Obesity energetics: Body weight regulation and the effects of diet composition. Gastroenterology.

[98] Tay J., Luscombe-Marsh N.D., Thompson C.H., Noakes M., Buckley J.D., Wittert G.A., Yancy W.S., Brinkworth G.D. (2015). Comparison of low- and high-carbohydrate diets for type 2 diabetes management: A randomized trial. Am. J. Clin. Nutr..

[99] Imamura F., Micha R., Wu J.H., de Oliveira Otto M.C., Otite F.O., Abioye A.I., Mozaffarian D. (2016). Effects of saturated fat, polyunsaturated fat, monounsaturated fat, and carbohydrate on glucose-insulin homeostasis: A systematic review and meta-analysis of randomised controlled feeding trials. PLoS Med..

[100] Dehghan M., Mente A., Zhang X., Swaminathan S., Li W., Mohan V., Iqbal R., Kumar R., Wentzel-Viljoen E., Rosengren A. (2017). Associations of fats and carbohydrate intake with cardiovascular disease and mortality in 18 countries from five continents (pure): A prospective cohort study. Lancet.

[101] Van Horn L., Carson J.A., Appel L.J., Burke L.E., Economos C., Karmally W., Lancaster K., Lichtenstein A.H., Johnson R.K., Thomas R.J. (2016). Recommended dietary pattern to achieve adherence to the american heart association/american college of cardiology (aha/acc) guidelines: A scientific statement from the american heart association. Circulation.

[102] Von Holstein-Rathlou S., BonDurant L.D., Peltekian L., Naber M.C., Yin T.C., Claflin K.E., Urizar A.I., Madsen A.N., Ratner C., Holst B. (2016). Fgf21 mediates endocrine control of simple sugar intake and sweet taste preference by the liver. Cell Metab..

[103] Talukdar S., Owen B.M., Song P., Hernandez G., Zhang Y., Zhou Y., Scott W.T., Paratala B., Turner T., Smith A. (2016). Fgf21 regulates sweet and alcohol preference. Cell Metab..

[104] Fisher F.M., Maratos-Flier E. (2016). Understanding the physiology of fgf21. Annu. Rev. Physiol..

[105] Soberg S., Sandholt C.H., Jespersen N.Z., Toft U., Madsen A.L., von Holstein-Rathlou S., Grevengoed T.J., Christensen K.B., Bredie W.L.P., Potthoff M.J. (2017). Fgf21 is a sugar-induced hormone associated with sweet intake and preference in humans. Cell Metab..

[106] Lundsgaard A.M., Fritzen A.M., Sjoberg K.A., Myrmel L.S., Madsen L., Wojtaszewski J.F., Richter E.A., Kiens B. (2017). Circulating fgf21 in humans is potently induced by short term overfeeding of carbohydrates. Mol. Metab..

[107] Shimbara T., Mondal M.S., Kawagoe T., Toshinai K., Koda S., Yamaguchi H., Date Y., Nakazato M. (2004). Central administration of ghrelin preferentially enhances fat ingestion. Neurosci. Lett..

[108] Schele E., Bake T., Rabasa C., Dickson S.L. (2016). Centrally administered ghrelin acutely influences food choice in rodents. PLoS ONE.

[109] Perello M., Sakata I., Birnbaum S., Chuang J.C., Osborne-Lawrence S., Rovinsky S.A., Woloszyn J., Yanagisawa M., Lutter M., Zigman J.M. (2010). Ghrelin increases the rewarding value of high-fat diet in an orexin-dependent manner. Biol. Psychiatry.

[110] Skibicka K.P., Hansson C., Egecioglu E., Dickson S.L. (2012). Role of ghrelin in food reward: Impact of ghrelin on sucrose self-administration and mesolimbic dopamine and acetylcholine receptor gene expression. Addict. Biol..

[111] Van der Klaauw A.A., Keogh J.M., Henning E., Stephenson C., Kelway S., Trowse V.M., Subramanian N., O’Rahilly S., Fletcher P.C., Farooqi I.S. (2016). Divergent effects of central melanocortin signalling on fat and sucrose preference in humans. Nat. Commun..

[112] Tung Y.C., Rimmington D., O’Rahilly S., Coll A.P. (2007). Pro-opiomelanocortin modulates the thermogenic and physical activity responses to high-fat feeding and markedly influences dietary fat preference. Endocrinology.

[113] Koegler F.H., Schaffhauser A.O., Mynatt R.L., York D.A., Bray G.A. (1999). Macronutrient diet intake of the lethal yellow agouti (ay/a) mouse. Physiol. Behav..

[114] Tempel D.L., Leibowitz K.J., Leibowitz S.F. (1988). Effects of pvn galanin on macronutrient selection. Peptides.

[115] Adams A.C., Clapham J.C., Wynick D., Speakman J.R. (2008). Feeding behaviour in galanin knockout mice supports a role of galanin in fat intake and preference. J. Neuroendocrinol..

[116] Karatayev O., Baylan J., Leibowitz S.F. (2009). Increased intake of ethanol and dietary fat in galanin overexpressing mice. Alcohol.

[117] Barson J.R., Morganstern I., Leibowitz S.F. (2010). Galanin and consummatory behavior: Special relationship with dietary fat, alcohol and circulating lipids. EXS.

[118] Leibowitz S.F., Akabayashi A., Wang J. (1998). Obesity on a high-fat diet: Role of hypothalamic galanin in neurons of the anterior paraventricular nucleus projecting to the median eminence. J. Neurosci..

[119] Wang J., Dourmashkin J.T., Yun R., Leibowitz S.F. (1999). Rapid changes in hypothalamic neuropeptide y produced by carbohydrate-rich meals that enhance corticosterone and glucose levels. Brain Res..

[120] McCue D.L., Kasper J.M., Hommel J.D. (2017). Regulation of motivation for food by neuromedin u in the paraventricular nucleus and the dorsal raphe nucleus. Int. J. Obes. (Lond.).

[121] Benzon C.R., Johnson S.B., McCue D.L., Li D., Green T.A., Hommel J.D. (2014). Neuromedin u receptor 2 knockdown in the paraventricular nucleus modifies behavioral responses to obesogenic high-fat food and leads to increased body weight. Neuroscience.

[122] Stanley B.G., Daniel D.R., Chin A.S., Leibowitz S.F. (1985). Paraventricular nucleus injections of peptide yy and neuropeptide y preferentially enhance carbohydrate ingestion. Peptides.

[123] Slawecki C.J., Betancourt M., Walpole T., Ehlers C.L. (2000). Increases in sucrose consumption, but not ethanol consumption, following icv npy administration. Pharmacol. Biochem. Behav..

[124] Elbers C.C., de Kovel C.G., van der Schouw Y.T., Meijboom J.R., Bauer F., Grobbee D.E., Trynka G., van Vliet-Ostaptchouk J.V., Wijmenga C., Onland-Moret N.C. (2009). Variants in neuropeptide y receptor 1 and 5 are associated with nutrient-specific food intake and are under recent selection in europeans. PLoS ONE.

[125] Van den Heuvel J.K., Furman K., Gumbs M.C., Eggels L., Opland D.M., Land B.B., Kolk S.M., Narayanan N.S., Fliers E., Kalsbeek A. (2015). Neuropeptide y activity in the nucleus accumbens modulates feeding behavior and neuronal activity. Biol. Psychiatry.

[126] Grinevich V., Desarmenien M.G., Chini B., Tauber M., Muscatelli F. (2015). Ontogenesis of oxytocin pathways in the mammalian brain: Late maturation and psychosocial disorders. Front. Neuroanat..

[127] Klockars A., Levine A.S., Olszewski P.K. (2015). Central oxytocin and food intake: Focus on macronutrient-driven reward. Front. Endocrinol. (Lausanne).

[128] Amico J.A., Vollmer R.R., Cai H.M., Miedlar J.A., Rinaman L. (2005). Enhanced initial and sustained intake of sucrose solution in mice with an oxytocin gene deletion. Am. J. Physiol. Regul. Integr. Comp. Physiol..

[129] Miedlar J.A., Rinaman L., Vollmer R.R., Amico J.A. (2007). Oxytocin gene deletion mice overconsume palatable sucrose solution but not palatable lipid emulsions. Am. J. Physiol. Regul. Integr. Comp. Physiol..

[130] Sclafani A., Rinaman L., Vollmer R.R., Amico J.A. (2007). Oxytocin knockout mice demonstrate enhanced intake of sweet and nonsweet carbohydrate solutions. Am. J. Physiol. Regul. Integr. Comp. Physiol..

[131] Olszewski P.K., Klockars A., Olszewska A.M., Fredriksson R., Schioth H.B., Levine A.S. (2010). Molecular, immunohistochemical, and pharmacological evidence of oxytocin’s role as inhibitor of carbohydrate but not fat intake. Endocrinology.

[132] Herisson F.M., Brooks L.L., Waas J.R., Levine A.S., Olszewski P.K. (2014). Functional relationship between oxytocin and appetite for carbohydrates versus saccharin. Neuroreport.

[133] Mitchell M.D., Haynes P.J., Anderson A.B., Turnbull A.C. (1981). Plasma oxytocin concentrations during the menstrual cycle. Eur. J. Obstet. Gynecol. Reprod. Biol..

[134] Salonia A., Nappi R.E., Pontillo M., Daverio R., Smeraldi A., Briganti A., Fabbri F., Zanni G., Rigatti P., Montorsi F. (2005). Menstrual cycle-related changes in plasma oxytocin are relevant to normal sexual function in healthy women. Horm. Behav..

[135] Sutton A.K., Pei H., Burnett K.H., Myers M.G., Rhodes C.J., Olson D.P. (2014). Control of food intake and energy expenditure by nos1 neurons of the paraventricular hypothalamus. J. Neurosci..

[136] van der Klaauw A.A., Ziauddeen H., Keogh J.M., Henning E., Dachi S., Fletcher P.C., Farooqi I.S. (2017). Oxytocin administration suppresses hypothalamic activation in response to visual food cues. Sci. Rep..

[137] Mullis K., Kay K., Williams D.L. (2013). Oxytocin action in the ventral tegmental area affects sucrose intake. Brain Res..

[138] Herisson F.M., Waas J.R., Fredriksson R., Schioth H.B., Levine A.S., Olszewski P.K. (2016). Oxytocin acting in the nucleus accumbens core decreases food intake. J. Neuroendocrinol..

[139] Sinclair M.S., Perea-Martinez I., Dvoryanchikov G., Yoshida M., Nishimori K., Roper S.D., Chaudhari N. (2010). Oxytocin signaling in mouse taste buds. PLoS ONE.

[140] Sinclair M.S., Perea-Martinez I., Abouyared M., St John S.J., Chaudhari N. (2015). Oxytocin decreases sweet taste sensitivity in mice. Physiol. Behav..

[141] Sakamaki R., Uemoto M., Inui A., Asakawa A., Ueno N., Ishibashi C., Hirono S., Yukioka H., Kato A., Shinfuku N. (2005). Melanin-concentrating hormone enhances sucrose intake. Int. J. Mol. Med..

[142] Domingos A.I., Sordillo A., Dietrich M.O., Liu Z.W., Tellez L.A., Vaynshteyn J., Ferreira J.G., Ekstrand M.I., Horvath T.L., de Araujo I.E. (2013). Hypothalamic melanin concentrating hormone neurons communicate the nutrient value of sugar. eLife.

[143] Sclafani A., Adamantidis A., Ackroff K. (2016). Mch receptor deletion does not impair glucose-conditioned flavor preferences in mice. Physiol. Behav..

[144] Heinrichs S.C., Britton K.T., Koob G.F. (1991). Both conditioned taste preference and aversion induced by corticotropin-releasing factor. Pharmacol. Biochem. Behav..

[145] Kumar B.A., Papamichael M., Leibowitz S.F. (1988). Feeding and macronutrient selection patterns in rats: Adrenalectomy and chronic corticosterone replacement. Physiol. Behav..

[146] Kumar B.A., Leibowitz S.F. (1988). Impact of acute corticosterone administration on feeding and macronutrient self-selection patterns. Am. J. Physiol..

[147] Bligh M.E., Douglass L.W., Castonguay T.W. (1993). Corticosterone modulation of dietary selection patterns. Physiol. Behav..

[148] Teegarden S.L., Bale T.L. (2008). Effects of stress on dietary preference and intake are dependent on access and stress sensitivity. Physiol. Behav..

[149] Wang S.S., Yan X.B., Hofman M.A., Swaab D.F., Zhou J.N. (2010). Increased expression level of corticotropin-releasing hormone in the amygdala and in the hypothalamus in rats exposed to chronic unpredictable mild stress. Neurosci. Bull..

[150] Heinrichs S.C., Koob G.F. (1992). Corticotropin-releasing factor modulates dietary preference in nutritionally and physically stressed rats. Psychopharmacology (Berl).

[151] Hajnal A., Smith G.P., Norgren R. (2004). Oral sucrose stimulation increases accumbens dopamine in the rat. Am. J. Physiol. Regul. Integr. Comp. Physiol..

[152] Carleton A., Accolla R., Simon S.A. (2010). Coding in the mammalian gustatory system. Trends Neurosci..

[153] Li C.S., Chung S., Lu D.P., Cho Y.K. (2012). Descending projections from the nucleus accumbens shell suppress activity of taste-responsive neurons in the hamster parabrachial nuclei. J. Neurophysiol..

[154] Ren X., Ferreira J.G., Zhou L., Shammah-Lagnado S.J., Yeckel C.W., de Araujo I.E. (2010). Nutrient selection in the absence of taste receptor signaling. J. Neurosci..

[155] de Araujo I.E., Oliveira-Maia A.J., Sotnikova T.D., Gainetdinov R.R., Caron M.G., Nicolelis M.A., Simon S.A. (2008). Food reward in the absence of taste receptor signaling. Neuron.

[156] Delaere F., Akaoka H., De Vadder F., Duchampt A., Mithieux G. (2013). Portal glucose influences the sensory, cortical and reward systems in rats. Eur. J. Neurosci..

[157] Oliveira-Maia A.J., Roberts C.D., Walker Q.D., Luo B., Kuhn C., Simon S.A., Nicolelis M.A. (2011). Intravascular food reward. PLoS ONE.

[158] Karnani M., Burdakov D. (2011). Multiple hypothalamic circuits sense and regulate glucose levels. Am. J. Physiol. Regul. Integr. Comp. Physiol..

[159] Cansell C., Castel J., Denis R.G., Rouch C., Delbes A.S., Martinez S., Mestivier D., Finan B., Maldonado-Aviles J.G., Rijnsburger M. (2014). Dietary triglycerides act on mesolimbic structures to regulate the rewarding and motivational aspects of feeding. Mol. Psychiatry.

[160] Dela Cruz J.A., Icaza-Cukali D., Tayabali H., Sampson C., Galanopoulos V., Bamshad D., Touzani K., Sclafani A., Bodnar R.J. (2012). Roles of dopamine d1 and d2 receptors in the acquisition and expression of fat-conditioned flavor preferences in rats. Neurobiol. Learn. Mem..

[161] Hankir M.K., Seyfried F., Hintschich C.A., Diep T.A., Kleberg K., Kranz M., Deuther-Conrad W., Tellez L.A., Rullmann M., Patt M. (2017). Gastric bypass surgery recruits a gut ppar-alpha-striatal d1r pathway to reduce fat appetite in obese rats. Cell Metab..

[162] Liu Q., Tabuchi M., Liu S., Kodama L., Horiuchi W., Daniels J., Chiu L., Baldoni D., Wu M.N. (2017). Branch-specific plasticity of a bifunctional dopamine circuit encodes protein hunger. Science.

[163] Haghighi A., Melka M.G., Bernard M., Abrahamowicz M., Leonard G.T., Richer L., Perron M., Veillette S., Xu C.J., Greenwood C.M. (2014). Opioid receptor mu 1 gene, fat intake and obesity in adolescence. Mol. Psychiatry.

[164] Bhakthavatsalam P., Leibowitz S.F. (1986). Morphine-elicited feeding: Diurnal rhythm, circulating corticosterone and macronutrient selection. Pharmacol. Biochem. Behav..

[165] Zhang M., Gosnell B.A., Kelley A.E. (1998). Intake of high-fat food is selectively enhanced by mu opioid receptor stimulation within the nucleus accumbens. J. Pharmacol. Exp. Ther..

[166] Sakamoto K., Okahashi T., Matsumura S., Okafuji Y., Adachi S., Tsuzuki S., Inoue K., Fushiki T. (2014). The opioid system majorly contributes to preference for fat emulsions but not sucrose solutions in mice. Biosci. Biotechnol. Biochem..

[167] Sakamoto K., Matsumura S., Okafuji Y., Eguchi A., Yoneda T., Mizushige T., Tsuzuki S., Inoue K., Fushiki T. (2015). The opioid system contributes to the acquisition of reinforcement for dietary fat but is not required for its maintenance. Physiol. Behav..

[168] Hankir M.K., Patt M., Patt J.T., Becker G.A., Rullmann M., Kranz M., Deuther-Conrad W., Schischke K., Seyfried F., Brust P. (2017). Suppressed fat appetite after roux-en-y gastric bypass surgery associates with reduced brain mu-opioid receptor availability in diet-induced obese male rats. Front. Neurosci..

[169] Zhang D., Li Z., Wang H., Yang M., Liang L., Fu J., Wang C., Ling J., Zhang Y., Zhang S. (2015). Interactions between obesity-related copy number variants and dietary behaviors in childhood obesity. Nutrients.

[170] Khandekar N., Berning B.A., Sainsbury A., Lin S. (2015). The role of pancreatic polypeptide in the regulation of energy homeostasis. Mol. Cell. Endocrinol..

[171] Bauer F., Elbers C.C., Adan R.A., Loos R.J., Onland-Moret N.C., Grobbee D.E., van Vliet-Ostaptchouk J.V., Wijmenga C., van der Schouw Y.T. (2009). Obesity genes identified in genome-wide association studies are associated with adiposity measures and potentially with nutrient-specific food preference. Am. J. Clin. Nutr..

[172] Chu A.Y., Workalemahu T., Paynter N.P., Rose L.M., Giulianini F., Tanaka T., Ngwa J.S., Qi Q., Curhan G.C., Rimm E.B. (2013). Novel locus including fgf21 is associated with dietary macronutrient intake. Hum. Mol. Genet..

[173] Tanaka T., Ngwa J.S., van Rooij F.J., Zillikens M.C., Wojczynski M.K., Frazier-Wood A.C., Houston D.K., Kanoni S., Lemaitre R.N., Luan J. (2013). Genome-wide meta-analysis of observational studies shows common genetic variants associated with macronutrient intake. Am. J. Clin. Nutr..

[174] Frayling T.M., Timpson N.J., Weedon M.N., Zeggini E., Freathy R.M., Lindgren C.M., Perry J.R., Elliott K.S., Lango H., Rayner N.W. (2007). A common variant in the fto gene is associated with body mass index and predisposes to childhood and adult obesity. Science.

[175] Brunkwall L., Ericson U., Hellstrand S., Gullberg B., Orho-Melander M., Sonestedt E. (2013). Genetic variation in the fat mass and obesity-associated gene (fto) in association with food preferences in healthy adults. Food Nutr. Res..

[176] Steemburgo T., Azevedo M.J., Gross J.L., Milagro F.I., Campion J., Martinez J.A. (2013). The rs9939609 polymorphism in the fto gene is associated with fat and fiber intakes in patients with type 2 diabetes. J. Nutrigenet. Nutrigenomics.

[177] Sonestedt E., Roos C., Gullberg B., Ericson U., Wirfalt E., Orho-Melander M. (2009). Fat and carbohydrate intake modify the association between genetic variation in the fto genotype and obesity. Am. J. Clin. Nutr..

[178] Gerken T., Girard C.A., Tung Y.C., Webby C.J., Saudek V., Hewitson K.S., Yeo G.S., McDonough M.A., Cunliffe S., McNeill L.A. (2007). The obesity-associated fto gene encodes a 2-oxoglutarate-dependent nucleic acid demethylase. Science.

[179] Jia G., Fu Y., Zhao X., Dai Q., Zheng G., Yang Y., Yi C., Lindahl T., Pan T., Yang Y.G. (2011). N6-methyladenosine in nuclear rna is a major substrate of the obesity-associated fto. Nat. Chem. Biol..

[180] Mauer J., Luo X., Blanjoie A., Jiao X., Grozhik A.V., Patil D.P., Linder B., Pickering B.F., Vasseur J.J., Chen Q. (2017). Reversible methylation of m6am in the 5′ cap controls mrna stability. Nature.

[181] Olszewski P.K., Fredriksson R., Eriksson J.D., Mitra A., Radomska K.J., Gosnell B.A., Solvang M.N., Levine A.S., Schioth H.B. (2011). Fto colocalizes with a satiety mediator oxytocin in the brain and upregulates oxytocin gene expression. Biochem. Biophys. Res. Commun..

[182] Karra E., O’Daly O.G., Choudhury A.I., Yousseif A., Millership S., Neary M.T., Scott W.R., Chandarana K., Manning S., Hess M.E. (2013). A link between fto, ghrelin, and impaired brain food-cue responsivity. J. Clin. Investig..

[183] Ridaura V.K., Faith J.J., Rey F.E., Cheng J., Duncan A.E., Kau A.L., Griffin N.W., Lombard V., Henrissat B., Bain J.R. (2013). Gut microbiota from twins discordant for obesity modulate metabolism in mice. Science.

[184] Liu R., Hong J., Xu X., Feng Q., Zhang D., Gu Y., Shi J., Zhao S., Liu W., Wang X. (2017). Gut microbiome and serum metabolome alterations in obesity and after weight-loss intervention. Nat. Med..

[185] Buffington S.A., Di Prisco G.V., Auchtung T.A., Ajami N.J., Petrosino J.F., Costa-Mattioli M. (2016). Microbial reconstitution reverses maternal diet-induced social and synaptic deficits in offspring. Cell.

[186] Asher G., Sassone-Corsi P. (2015). Time for food: The intimate interplay between nutrition, metabolism, and the circadian clock. Cell.

[187] Bray M.S., Tsai J.Y., Villegas-Montoya C., Boland B.B., Blasier Z., Egbejimi O., Kueht M., Young M.E. (2010). Time-of-day-dependent dietary fat consumption influences multiple cardiometabolic syndrome parameters in mice. Int. J. Obes. (Lond.).

[188] McHill A.W., Melanson E.L., Higgins J., Connick E., Moehlman T.M., Stothard E.R., Wright K.P. (2014). Impact of circadian misalignment on energy metabolism during simulated nightshift work. Proc. Natl. Acad. Sci. USA.

[189] Scheer F.A., Hilton M.F., Mantzoros C.S., Shea S.A. (2009). Adverse metabolic and cardiovascular consequences of circadian misalignment. Proc. Natl. Acad. Sci. USA.

[190] Pan A., Schernhammer E.S., Sun Q., Hu F.B. (2011). Rotating night shift work and risk of type 2 diabetes: Two prospective cohort studies in women. PLoS Med..

[191] Timlin M.T., Pereira M.A., Story M., Neumark-Sztainer D. (2008). Breakfast eating and weight change in a 5-year prospective analysis of adolescents: Project eat (eating among teens). Pediatrics.

[192] Garaulet M., Gomez-Abellan P., Alburquerque-Bejar J.J., Lee Y.C., Ordovas J.M., Scheer F.A. (2013). Timing of food intake predicts weight loss effectiveness. Int. J. Obes. (Lond.).

[193] Jakubowicz D., Barnea M., Wainstein J., Froy O. (2013). High caloric intake at breakfast vs. Dinner differentially influences weight loss of overweight and obese women. Obesity (Silver Spring).

[194] Yoshizaki T., Tada Y., Hida A., Sunami A., Yokoyama Y., Yasuda J., Nakai A., Togo F., Kawano Y. (2013). Effects of feeding schedule changes on the circadian phase of the cardiac autonomic nervous system and serum lipid levels. Eur. J. Appl. Physiol..

[195] Colles S.L., Dixon J.B., O’Brien P.E. (2007). Night eating syndrome and nocturnal snacking: Association with obesity, binge eating and psychological distress. Int. J. Obes. (Lond.).

[196] Koopman K.E., Caan M.W., Nederveen A.J., Pels A., Ackermans M.T., Fliers E., la Fleur S.E., Serlie M.J. (2014). Hypercaloric diets with increased meal frequency, but not meal size, increase intrahepatic triglycerides: A randomized controlled trial. Hepatology.

[197] Zarrinpar A., Chaix A., Panda S. (2016). Daily eating patterns and their impact on health and disease. Trends Endocrinol. Metab..

[198] Turek F.W., Joshu C., Kohsaka A., Lin E., Ivanova G., McDearmon E., Laposky A., Losee-Olson S., Easton A., Jensen D.R. (2005). Obesity and metabolic syndrome in circadian clock mutant mice. Science.

[199] Kettner N.M., Mayo S.A., Hua J., Lee C., Moore D.D., Fu L. (2015). Circadian dysfunction induces leptin resistance in mice. Cell Metab..

[200] Fukagawa K., Sakata T., Yoshimatsu H., Fujimoto K., Uchimura K., Asano C. (1992). Advance shift of feeding circadian rhythm induced by obesity progression in zucker rats. Am. J. Physiol..

[201] Arble D.M., Bass J., Laposky A.D., Vitaterna M.H., Turek F.W. (2009). Circadian timing of food intake contributes to weight gain. Obesity (Silver Spring).

[202] Garaulet M., Gomez-Abellan P. (2014). Timing of food intake and obesity: A novel association. Physiol. Behav..

[203] Sherman H., Genzer Y., Cohen R., Chapnik N., Madar Z., Froy O. (2012). Timed high-fat diet resets circadian metabolism and prevents obesity. FASEB J..

[204] Hatori M., Vollmers C., Zarrinpar A., DiTacchio L., Bushong E.A., Gill S., Leblanc M., Chaix A., Joens M., Fitzpatrick J.A. (2012). Time-restricted feeding without reducing caloric intake prevents metabolic diseases in mice fed a high-fat diet. Cell Metab..

[205] Fuse Y., Hirao A., Kuroda H., Otsuka M., Tahara Y., Shibata S. (2012). Differential roles of breakfast only (one meal per day) and a bigger breakfast with a small dinner (two meals per day) in mice fed a high-fat diet with regard to induced obesity and lipid metabolism. J. Circadian Rhythms.

[206] Salgado-Delgado R., Angeles-Castellanos M., Saderi N., Buijs R.M., Escobar C. (2010). Food intake during the normal activity phase prevents obesity and circadian desynchrony in a rat model of night work. Endocrinology.

[207] Wu T., Sun L., ZhuGe F., Guo X., Zhao Z., Tang R., Chen Q., Chen L., Kato H., Fu Z. (2011). Differential roles of breakfast and supper in rats of a daily three-meal schedule upon circadian regulation and physiology. Chronobiol. Int..

[208] Yoshida C., Shikata N., Seki S., Koyama N., Noguchi Y. (2012). Early nocturnal meal skipping alters the peripheral clock and increases lipogenesis in mice. Nutr. Metab. (Lond.).

[209] Albrecht U. (2012). Timing to perfection: The biology of central and peripheral circadian clocks. Neuron.

[210] Partch C.L., Green C.B., Takahashi J.S. (2014). Molecular architecture of the mammalian circadian clock. Trends Cell Biol..

[211] Herzog E.D., Hermanstyne T., Smyllie N.J., Hastings M.H. (2017). Regulating the suprachiasmatic nucleus (scn) circadian clockwork: Interplay between cell-autonomous and circuit-level mechanisms. Cold Spring Harb. Perspect. Biol..

[212] Moore R.Y., Lenn N.J. (1972). A retinohypothalamic projection in the rat. J. Comp. Neurol..

[213] Hendrickson A.E., Wagoner N., Cowan W.M. (1972). An autoradiographic and electron microscopic study of retino-hypothalamic connections. Z. Zellforsch. Mikrosk. Anat..

[214] Berson D.M., Dunn F.A., Takao M. (2002). Phototransduction by retinal ganglion cells that set the circadian clock. Science.

[215] Panda S., Provencio I., Tu D.C., Pires S.S., Rollag M.D., Castrucci A.M., Pletcher M.T., Sato T.K., Wiltshire T., Andahazy M. (2003). Melanopsin is required for non-image-forming photic responses in blind mice. Science.

[216] Panda S., Sato T.K., Castrucci A.M., Rollag M.D., DeGrip W.J., Hogenesch J.B., Provencio I., Kay S.A. (2002). Melanopsin (opn4) requirement for normal light-induced circadian phase shifting. Science.

[217] Provencio I., Rodriguez I.R., Jiang G., Hayes W.P., Moreira E.F., Rollag M.D. (2000). A novel human opsin in the inner retina. J. Neurosci..

[218] Provencio I., Rollag M.D., Castrucci A.M. (2002). Photoreceptive net in the mammalian retina. This mesh of cells may explain how some blind mice can still tell day from night. Nature.

[219] Morin L.P. (2013). Neuroanatomy of the extended circadian rhythm system. Exp. Neurol..

[220] Luo A.H., Aston-Jones G. (2009). Circuit projection from suprachiasmatic nucleus to ventral tegmental area: A novel circadian output pathway. Eur. J. Neurosci..

[221] Moorman D.E., Aston-Jones G. (2010). Orexin/hypocretin modulates response of ventral tegmental dopamine neurons to prefrontal activation: Diurnal influences. J. Neurosci..

[222] Bourdy R., Barrot M. (2012). A new control center for dopaminergic systems: Pulling the vta by the tail. Trends Neurosci..

[223] Aston-Jones G., Chen S., Zhu Y., Oshinsky M.L. (2001). A neural circuit for circadian regulation of arousal. Nat. Neurosci..

[224] Chou T.C., Scammell T.E., Gooley J.J., Gaus S.E., Saper C.B., Lu J. (2003). Critical role of dorsomedial hypothalamic nucleus in a wide range of behavioral circadian rhythms. J. Neurosci..

[225] Buijs R.M., Hou Y.X., Shinn S., Renaud L.P. (1994). Ultrastructural evidence for intra- and extranuclear projections of gabaergic neurons of the suprachiasmatic nucleus. J. Comp. Neurol..

[226] Vrang N., Larsen P.J., Mikkelsen J.D. (1995). Direct projection from the suprachiasmatic nucleus to hypophysiotrophic corticotropin-releasing factor immunoreactive cells in the paraventricular nucleus of the hypothalamus demonstrated by means of phaseolus vulgaris-leucoagglutinin tract tracing. Brain Res..

[227] Abrahamson E.E., Leak R.K., Moore R.Y. (2001). The suprachiasmatic nucleus projects to posterior hypothalamic arousal systems. Neuroreport.

[228] Schibler U., Ripperger J., Brown S.A. (2003). Peripheral circadian oscillators in mammals: Time and food. J. Biol. Rhythms.

[229] Vollmers C., Gill S., DiTacchio L., Pulivarthy S.R., Le H.D., Panda S. (2009). Time of feeding and the intrinsic circadian clock drive rhythms in hepatic gene expression. Proc. Natl. Acad. Sci. USA.

[230] Eckel-Mahan K.L., Patel V.R., de Mateo S., Orozco-Solis R., Ceglia N.J., Sahar S., Dilag-Penilla S.A., Dyar K.A., Baldi P., Sassone-Corsi P. (2013). Reprogramming of the circadian clock by nutritional challenge. Cell.

[231] Buijs R.M., la Fleur S.E., Wortel J., Van Heyningen C., Zuiddam L., Mettenleiter T.C., Kalsbeek A., Nagai K., Niijima A. (2003). The suprachiasmatic nucleus balances sympathetic and parasympathetic output to peripheral organs through separate preautonomic neurons. J. Comp. Neurol..

[232] Cheng M.Y., Bullock C.M., Li C., Lee A.G., Bermak J.C., Belluzzi J., Weaver D.R., Leslie F.M., Zhou Q.Y. (2002). Prokineticin 2 transmits the behavioural circadian rhythm of the suprachiasmatic nucleus. Nature.

[233] Li J.D., Hu W.P., Boehmer L., Cheng M.Y., Lee A.G., Jilek A., Siegel J.M., Zhou Q.Y. (2006). Attenuated circadian rhythms in mice lacking the prokineticin 2 gene. J. Neurosci..

[234] Prosser H.M., Bradley A., Chesham J.E., Ebling F.J., Hastings M.H., Maywood E.S. (2007). Prokineticin receptor 2 (prokr2) is essential for the regulation of circadian behavior by the suprachiasmatic nuclei. Proc. Natl. Acad. Sci. USA.

[235] Kalsbeek A., Buijs R.M., van Heerikhuize J.J., Arts M., van der Woude T.P. (1992). Vasopressin-containing neurons of the suprachiasmatic nuclei inhibit corticosterone release. Brain Res..

[236] Kalsbeek A., Fliers E., Hofman M.A., Swaab D.F., Buijs R.M. (2010). Vasopressin and the output of the hypothalamic biological clock. J. Neuroendocrinol..

[237] Kraves S., Weitz C.J. (2006). A role for cardiotrophin-like cytokine in the circadian control of mammalian locomotor activity. Nat. Neurosci..

[238] Kalsbeek A., Buijs R.M. (1992). Peptidergic transmitters of the suprachiasmatic nuclei and the control of circadian rhythmicity. Prog. Brain Res..

[239] Kantor S., Mochizuki T., Janisiewicz A.M., Clark E., Nishino S., Scammell T.E. (2009). Orexin neurons are necessary for the circadian control of rem sleep. Sleep.

[240] Mieda M., Williams S.C., Sinton C.M., Richardson J.A., Sakurai T., Yanagisawa M. (2004). Orexin neurons function in an efferent pathway of a food-entrainable circadian oscillator in eliciting food-anticipatory activity and wakefulness. J. Neurosci..

[241] Webb I.C., Patton D.F., Hamson D.K., Mistlberger R.E. (2008). Neural correlates of arousal-induced circadian clock resetting: Hypocretin/orexin and the intergeniculate leaflet. Eur. J. Neurosci..

[242] Colwell C.S., Michel S., Itri J., Rodriguez W., Tam J., Lelievre V., Hu Z., Waschek J.A. (2004). Selective deficits in the circadian light response in mice lacking pacap. Am. J. Physiol. Regul. Integr. Comp. Physiol..

[243] Hannibal J., Georg B., Fahrenkrug J. (2016). Altered circadian food anticipatory activity rhythms in pacap receptor 1 (pac1) deficient mice. PLoS ONE.

[244] Kawaguchi C., Tanaka K., Isojima Y., Shintani N., Hashimoto H., Baba A., Nagai K. (2003). Changes in light-induced phase shift of circadian rhythm in mice lacking pacap. Biochem. Biophys. Res. Commun..

[245] Li X., Sankrithi N., Davis F.C. (2002). Transforming growth factor-alpha is expressed in astrocytes of the suprachiasmatic nucleus in hamster: Role of glial cells in circadian clocks. Neuroreport.

[246] Engel L., Lorenzkowski V., Langer C., Rohleder N., Spessert R. (2005). The photoperiod entrains the molecular clock of the rat pineal. Eur. J. Neurosci..

[247] Moore R.Y. (1996). Neural control of the pineal gland. Behav. Brain Res..

[248] Kalsbeek A., van der Vliet J., Buijs R.M. (1996). Decrease of endogenous vasopressin release necessary for expression of the circadian rise in plasma corticosterone: A reverse microdialysis study. J. Neuroendocrinol..

[249] Kalsbeek A., van Heerikhuize J.J., Wortel J., Buijs R.M. (1996). A diurnal rhythm of stimulatory input to the hypothalamo-pituitary-adrenal system as revealed by timed intrahypothalamic administration of the vasopressin v1 antagonist. J. Neurosci..

[250] Boulos Z., Rosenwasser A.M., Terman M. (1980). Feeding schedules and the circadian organization of behavior in the rat. Behav. Brain Res..

[251] Stephan F.K., Swann J.M., Sisk C.L. (1979). Anticipation of 24-hr feeding schedules in rats with lesions of the suprachiasmatic nucleus. Behav. Neural Biol..

[252] Honma K., Honma S. (2009). The scn-independent clocks, methamphetamine and food restriction. Eur. J. Neurosci..

[253] Mendoza J., Pevet P., Felder-Schmittbuhl M.P., Bailly Y., Challet E. (2010). The cerebellum harbors a circadian oscillator involved in food anticipation. J. Neurosci..

[254] Landry G.J., Kent B.A., Patton D.F., Jaholkowski M., Marchant E.G., Mistlberger R.E. (2011). Evidence for time-of-day dependent effect of neurotoxic dorsomedial hypothalamic lesions on food anticipatory circadian rhythms in rats. PLoS ONE.

[255] Mieda M., Williams S.C., Richardson J.A., Tanaka K., Yanagisawa M. (2006). The dorsomedial hypothalamic nucleus as a putative food-entrainable circadian pacemaker. Proc. Natl. Acad. Sci. USA.

[256] Gooley J.J., Schomer A., Saper C.B. (2006). The dorsomedial hypothalamic nucleus is critical for the expression of food-entrainable circadian rhythms. Nat. Neurosci..

[257] Gallardo C.M., Darvas M., Oviatt M., Chang C.H., Michalik M., Huddy T.F., Meyer E.E., Shuster S.A., Aguayo A., Hill E.M. (2014). Dopamine receptor 1 neurons in the dorsal striatum regulate food anticipatory circadian activity rhythms in mice. eLife.

[258] Verwey M., Amir S. (2009). Food-entrainable circadian oscillators in the brain. Eur. J. Neurosci..

[259] Mendoza J., Angeles-Castellanos M., Escobar C. (2005). Entrainment by a palatable meal induces food-anticipatory activity and c-fos expression in reward-related areas of the brain. Neuroscience.

[260] Bechtold D.A., Loudon A.S. (2013). Hypothalamic clocks and rhythms in feeding behaviour. Trends Neurosci..

[261] Sutton G.M., Perez-Tilve D., Nogueiras R., Fang J., Kim J.K., Cone R.D., Gimble J.M., Tschop M.H., Butler A.A. (2008). The melanocortin-3 receptor is required for entrainment to meal intake. J. Neurosci..

[262] Landry G.J., Yamakawa G.R., Webb I.C., Mear R.J., Mistlberger R.E. (2007). The dorsomedial hypothalamic nucleus is not necessary for the expression of circadian food-anticipatory activity in rats. J. Biol. Rhythms.

[263] Orozco-Solis R., Ramadori G., Coppari R., Sassone-Corsi P. (2015). Sirt1 relays nutritional inputs to the circadian clock through the sf1 neurons of the ventromedial hypothalamus. Endocrinology.

[264] Orozco-Solis R., Aguilar-Arnal L., Murakami M., Peruquetti R., Ramadori G., Coppari R., Sassone-Corsi P. (2016). The circadian clock in the ventromedial hypothalamus controls cyclic energy expenditure. Cell Metab..

[265] Sasaki T., Kikuchi O., Shimpuku M., Susanti V.Y., Yokota-Hashimoto H., Taguchi R., Shibusawa N., Sato T., Tang L., Amano K. (2014). Hypothalamic sirt1 prevents age-associated weight gain by improving leptin sensitivity in mice. Diabetologia.

[266] Sasaki T., Kim H.J., Kobayashi M., Kitamura Y.I., Yokota-Hashimoto H., Shiuchi T., Minokoshi Y., Kitamura T. (2010). Induction of hypothalamic sirt1 leads to cessation of feeding via agouti-related peptide. Endocrinology.

[267] Satoh A., Brace C.S., Ben-Josef G., West T., Wozniak D.F., Holtzman D.M., Herzog E.D., Imai S. (2010). Sirt1 promotes the central adaptive response to diet restriction through activation of the dorsomedial and lateral nuclei of the hypothalamus. J. Neurosci..

[268] Chen D., Steele A.D., Lindquist S., Guarente L. (2005). Increase in activity during calorie restriction requires sirt1. Science.

[269] Mendoza J., Clesse D., Pevet P., Challet E. (2010). Food-reward signalling in the suprachiasmatic clock. J. Neurochem..

[270] Smit A.N., Patton D.F., Michalik M., Opiol H., Mistlberger R.E. (2013). Dopaminergic regulation of circadian food anticipatory activity rhythms in the rat. PLoS ONE.

[271] Mieda M., Sakurai T. (2011). Bmal1 in the nervous system is essential for normal adaptation of circadian locomotor activity and food intake to periodic feeding. J. Neurosci..

[272] Pendergast J.S., Oda G.A., Niswender K.D., Yamazaki S. (2012). Period determination in the food-entrainable and methamphetamine-sensitive circadian oscillator(s). Proc. Natl. Acad. Sci. USA.

[273] Pitts S., Perone E., Silver R. (2003). Food-entrained circadian rhythms are sustained in arrhythmic clk/clk mutant mice. Am. J. Physiol. Regul. Integr. Comp. Physiol..

[274] Dudley C.A., Erbel-Sieler C., Estill S.J., Reick M., Franken P., Pitts S., McKnight S.L. (2003). Altered patterns of sleep and behavioral adaptability in npas2-deficient mice. Science.

[275] Iijima M., Yamaguchi S., van der Horst G.T., Bonnefont X., Okamura H., Shibata S. (2005). Altered food-anticipatory activity rhythm in cryptochrome-deficient mice. Neurosci. Res..

[276] Van der Zee E.A., Havekes R., Barf R.P., Hut R.A., Nijholt I.M., Jacobs E.H., Gerkema M.P. (2008). Circadian time-place learning in mice depends on cry genes. Curr. Biol..

[277] Pendergast J.S., Nakamura W., Friday R.C., Hatanaka F., Takumi T., Yamazaki S. (2009). Robust food anticipatory activity in bmal1-deficient mice. PLoS ONE.

[278] Storch K.F., Weitz C.J. (2009). Daily rhythms of food-anticipatory behavioral activity do not require the known circadian clock. Proc. Natl. Acad. Sci. USA.

[279] Mistlberger R., Rusak B. (1987). Palatable daily meals entrain anticipatory activity rhythms in free-feeding rats: Dependence on meal size and nutrient content. Physiol. Behav..

[280] Webb I.C., Baltazar R.M., Lehman M.N., Coolen L.M. (2009). Bidirectional interactions between the circadian and reward systems: Is restricted food access a unique zeitgeber?. Eur. J. Neurosci..

[281] Pendergast J.S., Branecky K.L., Yang W., Ellacott K.L., Niswender K.D., Yamazaki S. (2013). High-fat diet acutely affects circadian organisation and eating behavior. Eur. J. Neurosci..

[282] Webb I.C., Lehman M.N., Coolen L.M. (2015). Diurnal and circadian regulation of reward-related neurophysiology and behavior. Physiol. Behav..

[283] Chung S., Lee E.J., Yun S., Choe H.K., Park S.B., Son H.J., Kim K.S., Dluzen D.E., Lee I., Hwang O. (2014). Impact of circadian nuclear receptor rev-erbalpha on midbrain dopamine production and mood regulation. Cell.

[284] Webb I.C., Baltazar R.M., Wang X., Pitchers K.K., Coolen L.M., Lehman M.N. (2009). Diurnal variations in natural and drug reward, mesolimbic tyrosine hydroxylase, and clock gene expression in the male rat. J. Biol. Rhythms.

[285] Smith A.D., Olson R.J., Justice J.B. (1992). Quantitative microdialysis of dopamine in the striatum: Effect of circadian variation. J. Neurosci. Methods.

[286] Paulson P.E., Robinson T.E. (1994). Relationship between circadian changes in spontaneous motor activity and dorsal versus ventral striatal dopamine neurotransmission assessed with on-line microdialysis. Behav. Neurosci..

[287] Hood S., Cassidy P., Cossette M.P., Weigl Y., Verwey M., Robinson B., Stewart J., Amir S. (2010). Endogenous dopamine regulates the rhythm of expression of the clock protein per2 in the rat dorsal striatum via daily activation of d2 dopamine receptors. J. Neurosci..

[288] Ferris M.J., Espana R.A., Locke J.L., Konstantopoulos J.K., Rose J.H., Chen R., Jones S.R. (2014). Dopamine transporters govern diurnal variation in extracellular dopamine tone. Proc. Natl. Acad. Sci. USA.

[289] Castaneda T.R., de Prado B.M., Prieto D., Mora F. (2004). Circadian rhythms of dopamine, glutamate and gaba in the striatum and nucleus accumbens of the awake rat: Modulation by light. J. Pineal Res..

[290] O’Neill R.D., Fillenz M. (1985). Simultaneous monitoring of dopamine release in rat frontal cortex, nucleus accumbens and striatum: Effect of drugs, circadian changes and correlations with motor activity. Neuroscience.

[291] O’Neill R.D. (1990). Uric acid levels and dopamine transmission in rat striatum: Diurnal changes and effects of drugs. Brain Res..

[292] Hampp G., Ripperger J.A., Houben T., Schmutz I., Blex C., Perreau-Lenz S., Brunk I., Spanagel R., Ahnert-Hilger G., Meijer J.H. (2008). Regulation of monoamine oxidase a by circadian-clock components implies clock influence on mood. Curr. Biol..

[293] Baltazar R.M., Coolen L.M., Webb I.C. (2013). Diurnal rhythms in neural activation in the mesolimbic reward system: Critical role of the medial prefrontal cortex. Eur. J. Neurosci..

[294] Sleipness E.P., Sorg B.A., Jansen H.T. (2007). Diurnal differences in dopamine transporter and tyrosine hydroxylase levels in rat brain: Dependence on the suprachiasmatic nucleus. Brain Res..

[295] Mendoza J., Challet E. (2014). Circadian insights into dopamine mechanisms. Neuroscience.

[296] Mukherjee S., Coque L., Cao J.L., Kumar J., Chakravarty S., Asaithamby A., Graham A., Gordon E., Enwright J.F., DiLeone R.J. (2010). Knockdown of clock in the ventral tegmental area through rna interference results in a mixed state of mania and depression-like behavior. Biol. Psychiatry.

[297] Strother W.N., Norman A.B., Lehman M.N. (1998). D1-dopamine receptor binding and tyrosine hydroxylase-immunoreactivity in the fetal and neonatal hamster suprachiasmatic nucleus. Brain Res. Dev. Brain Res..

[298] Rivkees S.A., Lachowicz J.E. (1997). Functional d1 and d5 dopamine receptors are expressed in the suprachiasmatic, supraoptic, and paraventricular nuclei of primates. Synapse.

[299] Smyllie N.J., Chesham J.E., Hamnett R., Maywood E.S., Hastings M.H. (2016). Temporally chimeric mice reveal flexibility of circadian period-setting in the suprachiasmatic nucleus. Proc. Natl. Acad. Sci. USA.

[300] Honma S., Honma K., Hiroshige T. (1989). Methamphetamine induced locomotor rhythm entrains to restricted daily feeding in scn lesioned rats. Physiol. Behav..

[301] Ono M., Watanabe A., Matsumoto Y., Fukushima T., Nishikawa Y., Moriya T., Shibata S., Watanabe S. (1996). Methamphetamine modifies the photic entraining responses in the rodent suprachiasmatic nucleus via serotonin release. Neuroscience.

[302] Cohen S., Vainer E., Matar M.A., Kozlovsky N., Kaplan Z., Zohar J., Mathe A.A., Cohen H. (2015). Diurnal fluctuations in hpa and neuropeptide y-ergic systems underlie differences in vulnerability to traumatic stress responses at different zeitgeber times. Neuropsychopharmacology.

[303] Akabayashi A., Levin N., Paez X., Alexander J.T., Leibowitz S.F. (1994). Hypothalamic neuropeptide y and its gene expression: Relation to light/dark cycle and circulating corticosterone. Mol. Cell. Neurosci..

[304] Xu B., Kalra P.S., Farmerie W.G., Kalra S.P. (1999). Daily changes in hypothalamic gene expression of neuropeptide y, galanin, proopiomelanocortin, and adipocyte leptin gene expression and secretion: Effects of food restriction. Endocrinology.

[305] Yoshihara T., Honma S., Honma K. (1996). Effects of restricted daily feeding on neuropeptide y release in the rat paraventricular nucleus. Am. J. Physiol..

[306] Wiater M.F., Mukherjee S., Li A.J., Dinh T.T., Rooney E.M., Simasko S.M., Ritter S. (2011). Circadian integration of sleep-wake and feeding requires npy receptor-expressing neurons in the mediobasal hypothalamus. Am. J. Physiol. Regul. Integr. Comp. Physiol..

[307] Gunapala K.M., Gallardo C.M., Hsu C.T., Steele A.D. (2011). Single gene deletions of orexin, leptin, neuropeptide y, and ghrelin do not appreciably alter food anticipatory activity in mice. PLoS ONE.

[308] Yi C.X., van der Vliet J., Dai J., Yin G., Ru L., Buijs R.M. (2006). Ventromedial arcuate nucleus communicates peripheral metabolic information to the suprachiasmatic nucleus. Endocrinology.

[309] Card J.P., Moore R.Y. (1982). Ventral lateral geniculate nucleus efferents to the rat suprachiasmatic nucleus exhibit avian pancreatic polypeptide-like immunoreactivity. J. Comp. Neurol..

[310] Saderi N., Cazarez-Marquez F., Buijs F.N., Salgado-Delgado R.C., Guzman-Ruiz M.A., del Carmen Basualdo M., Escobar C., Buijs R.M. (2013). The npy intergeniculate leaflet projections to the suprachiasmatic nucleus transmit metabolic conditions. Neuroscience.

[311] Li A.J., Wiater M.F., Oostrom M.T., Smith B.R., Wang Q., Dinh T.T., Roberts B.L., Jansen H.T., Ritter S. (2012). Leptin-sensitive neurons in the arcuate nuclei contribute to endogenous feeding rhythms. Am. J. Physiol. Regul. Integr. Comp. Physiol..

[312] Tiesjema B., Adan R.A., Luijendijk M.C., Kalsbeek A., la Fleur S.E. (2007). Differential effects of recombinant adeno-associated virus-mediated neuropeptide y overexpression in the hypothalamic paraventricular nucleus and lateral hypothalamus on feeding behavior. J. Neurosci..

[313] Tuomisto L., Lozeva V., Valjakka A., Lecklin A. (2001). Modifying effects of histamine on circadian rhythms and neuronal excitability. Behav. Brain Res..

[314] Yu X., Zecharia A., Zhang Z., Yang Q., Yustos R., Jager P., Vyssotski A.L., Maywood E.S., Chesham J.E., Ma Y. (2014). Circadian factor bmal1 in histaminergic neurons regulates sleep architecture. Curr. Biol..

[315] Yoshimatsu H. (2008). Hypothalamic neuronal histamine regulates body weight through the modulation of diurnal feeding rhythm. Nutrition.

[316] Kohsaka A., Laposky A.D., Ramsey K.M., Estrada C., Joshu C., Kobayashi Y., Turek F.W., Bass J. (2007). High-fat diet disrupts behavioral and molecular circadian rhythms in mice. Cell Metab..

[317] Mendoza J., Pevet P., Challet E. (2008). High-fat feeding alters the clock synchronization to light. J. Physiol..

[318] Branecky K.L., Niswender K.D., Pendergast J.S. (2015). Disruption of daily rhythms by high-fat diet is reversible. PLoS ONE.

[319] Mifune H., Tajiri Y., Nishi Y., Hara K., Iwata S., Tokubuchi I., Mitsuzono R., Yamada K., Kojima M. (2015). Voluntary exercise contributed to an amelioration of abnormal feeding behavior, locomotor activity and ghrelin production concomitantly with a weight reduction in high fat diet-induced obese rats. Peptides.

[320] Blancas-Velazquez A., Mendoza J., Garcia A.N., la Fleur S.E. (2017). Diet-induced obesity and circadian disruption of feeding behavior. Front. Neurosci..

[321] Guan Z., Vgontzas A.N., Bixler E.O., Fang J. (2008). Sleep is increased by weight gain and decreased by weight loss in mice. Sleep.

[322] Jenkins J.B., Omori T., Guan Z., Vgontzas A.N., Bixler E.O., Fang J. (2006). Sleep is increased in mice with obesity induced by high-fat food. Physiol. Behav..

[323] Luppi M., Cerri M., Martelli D., Tupone D., Del Vecchio F., Di Cristoforo A., Perez E., Zamboni G., Amici R. (2014). Waking and sleeping in the rat made obese through a high-fat hypercaloric diet. Behav. Brain Res..

[324] Thaler J.P., Yi C.X., Schur E.A., Guyenet S.J., Hwang B.H., Dietrich M.O., Zhao X., Sarruf D.A., Izgur V., Maravilla K.R. (2012). Obesity is associated with hypothalamic injury in rodents and humans. J. Clin. Investig..

[325] Posey K.A., Clegg D.J., Printz R.L., Byun J., Morton G.J., Vivekanandan-Giri A., Pennathur S., Baskin D.G., Heinecke J.W., Woods S.C. (2009). Hypothalamic proinflammatory lipid accumulation, inflammation, and insulin resistance in rats fed a high-fat diet. Am. J. Physiol. Endocrinol. Metab..

[326] Borg M.L., Omran S.F., Weir J., Meikle P.J., Watt M.J. (2012). Consumption of a high-fat diet, but not regular endurance exercise training, regulates hypothalamic lipid accumulation in mice. J. Physiol..

[327] Valdearcos M., Robblee M.M., Benjamin D.I., Nomura D.K., Xu A.W., Koliwad S.K. (2014). Microglia dictate the impact of saturated fat consumption on hypothalamic inflammation and neuronal function. Cell Rep..

[328] Milanski M., Degasperi G., Coope A., Morari J., Denis R., Cintra D.E., Tsukumo D.M., Anhe G., Amaral M.E., Takahashi H.K. (2009). Saturated fatty acids produce an inflammatory response predominantly through the activation of tlr4 signaling in hypothalamus: Implications for the pathogenesis of obesity. J. Neurosci..

[329] Zhang X., Zhang G., Zhang H., Karin M., Bai H., Cai D. (2008). Hypothalamic ikkbeta/nf-kappab and er stress link overnutrition to energy imbalance and obesity. Cell.

[330] Valdearcos M., Douglass J.D., Robblee M.M., Dorfman M.D., Stifler D.R., Bennett M.L., Gerritse I., Fasnacht R., Barres B.A., Thaler J.P. (2017). Microglial inflammatory signaling orchestrates the hypothalamic immune response to dietary excess and mediates obesity susceptibility. Cell Metab..

[331] Douglass J.D., Dorfman M.D., Fasnacht R., Shaffer L.D., Thaler J.P. (2017). Astrocyte ikkbeta/nf-kappab signaling is required for diet-induced obesity and hypothalamic inflammation. Mol. Metab..

[332] Cai D. (2012). One step from prediabetes to diabetes: Hypothalamic inflammation?. Endocrinology.

[333] Jais A., Bruning J.C. (2017). Hypothalamic inflammation in obesity and metabolic disease. J. Clin. Investig..

[334] Morales L., Del Olmo N., Valladolid-Acebes I., Fole A., Cano V., Merino B., Stucchi P., Ruggieri D., Lopez L., Alguacil L.F. (2012). Shift of circadian feeding pattern by high-fat diets is coincident with reward deficits in obese mice. PLoS ONE.

[335] Cunningham P.S., Ahern S.A., Smith L.C., da Silva Santos C.S., Wager T.T., Bechtold D.A. (2016). Targeting of the circadian clock via ck1delta/epsilon to improve glucose homeostasis in obesity. Sci. Rep..

[336] Guilding C., Hughes A.T., Brown T.M., Namvar S., Piggins H.D. (2009). A riot of rhythms: Neuronal and glial circadian oscillators in the mediobasal hypothalamus. Mol. Brain.

[337] Oosterman J.E., Kalsbeek A., la Fleur S.E., Belsham D.D. (2015). Impact of nutrients on circadian rhythmicity. Am. J. Physiol. Regul. Integr. Comp. Physiol..

[338] Kentish S.J., Vincent A.D., Kennaway D.J., Wittert G.A., Page A.J. (2016). High-fat diet-induced obesity ablates gastric vagal afferent circadian rhythms. J. Neurosci..

[339] Del Rio D., Stucchi P., Hernandez-Nuno F., Cano V., Morales L., Chowen J.A., Del Olmo N., Ruiz-Gayo M. (2017). Free-choice high-fat diet alters circadian oscillation of energy intake in adolescent mice: Role of prefrontal cortex. Eur. J. Nutr..

[340] La Fleur S.E., Luijendijk M.C., van der Zwaal E.M., Brans M.A., Adan R.A. (2013). The snacking rat as model of human obesity: Effects of a free-choice high-fat high-sugar diet on meal patterns. Int. J. Obes. (Lond.).

